# Proteomic Profiling of Human Prostate Cancer-associated Fibroblasts (CAF) Reveals LOXL2-dependent Regulation of the Tumor Microenvironment*[Fn FN2]

**DOI:** 10.1074/mcp.RA119.001496

**Published:** 2019-05-06

**Authors:** Elizabeth V. Nguyen, Brooke A. Pereira, Mitchell G. Lawrence, Xiuquan Ma, Richard J. Rebello, Howard Chan, Birunthi Niranjan, Yunjian Wu, Stuart Ellem, Xiaoqing Guan, Jianmin Wu, Joanna N. Skhinas, Thomas R. Cox, Gail P. Risbridger, Renea A. Taylor, Natalie L. Lister, Roger J. Daly

**Affiliations:** From the ‡Cancer Program, Biomedicine Discovery Institute,; Departments of §Biochemistry and Molecular Biology,; ¶Anatomy and Developmental Biology, and; ‡‡‡Physiology, Monash University, Clayton, Australia;; ‖Cancer Research Division, Peter MacCallum Cancer Centre, Victorian Comprehensive Cancer Centre, Parkville, Australia;; **School of Health and Wellbeing, University of Southern Queensland, Ipswich, Queensland, Australia;; ‡‡Key Laboratory of Carcinogenesis and Translational Research (Ministry of Education/Beijing), Center for Cancer Bioinformatics, Peking University Cancer Hospital & Institute, Beijing, China;; §§The Garvan Institute of Medical Research and The Kinghorn Cancer Centre, Sydney, Australia;; ¶¶St Vincent's Clinical School, Faculty of Medicine, UNSW Sydney, Sydney, Australia;; ‖‖Sir Peter MacCallum Department of Oncology, The University of Melbourne, Parkville, Australia

**Keywords:** Phosphoproteome, Prostate cancer, Prostate cancer biomarkers, Tumor microenvironment*, Cancer biomarker(s), Cancer-associated fibroblasts, Fibroblasts, LOXL2, Non-malignant prostate fibroblasts

## Abstract

In prostate cancer, cancer-associated fibroblasts (CAF) exhibit contrasting biological properties to non-malignant prostate fibroblasts (NPF) and promote tumorigenesis. Resolving intercellular signaling pathways between CAF and prostate tumor epithelium may offer novel opportunities for research translation. To this end, the proteome and phosphoproteome of four pairs of patient-matched CAF and NPF were characterized to identify discriminating proteomic signatures. Samples were analyzed by liquid chromatography-tandem mass spectrometry (LC-MS/MS) with a hyper reaction monitoring data-independent acquisition (HRM-DIA) workflow. Proteins that exhibited a significant increase in CAF *versus* NPF were enriched for the functional categories “cell adhesion” and the “extracellular matrix.” The CAF phosphoproteome exhibited enhanced phosphorylation of proteins associated with the “spliceosome” and “actin binding.” STRING analysis of the CAF proteome revealed a prominent interaction hub associated with collagen synthesis, modification, and signaling. It contained multiple collagens, including the fibrillar types COL1A1/2 and COL5A1; the receptor tyrosine kinase discoidin domain-containing receptor 2 (DDR2), a receptor for fibrillar collagens; and lysyl oxidase-like 2 (LOXL2), an enzyme that promotes collagen crosslinking. Increased activity and/or expression of LOXL2 and DDR2 in CAF were confirmed by enzymatic assays and Western blotting analyses. Pharmacological inhibition of CAF-derived LOXL2 perturbed extracellular matrix (ECM) organization and decreased CAF migration in a wound healing assay. Further, it significantly impaired the motility of co-cultured RWPE-2 prostate tumor epithelial cells. These results indicate that CAF-derived LOXL2 is an important mediator of intercellular communication within the prostate tumor microenvironment and is a potential therapeutic target.

## Abstract (precis)

Mass spectrometry-based (phospho)proteomics identified a prominent hub associated with collagens, receptor tyrosine kinase discoidin domain-containing receptor 2 (DDR2), and lysyl oxidase-like 2 (LOXL2) from patient-matched cancer-associated fibroblasts (CAF) and non-malignant prostate fibroblasts (NPF). The functional role of LOXL2 in regulating ECM organization and migration of both CAF and co-cultured prostate cancer cells was validated with LOXL2 inhibitors. Our data provide the first demonstration that prostate CAF-dependent LOXL2 production controls prostate tumor cell motility, highlighting LOXL2 as an attractive therapeutic target.

## Graphical Abstract

**Figure:**
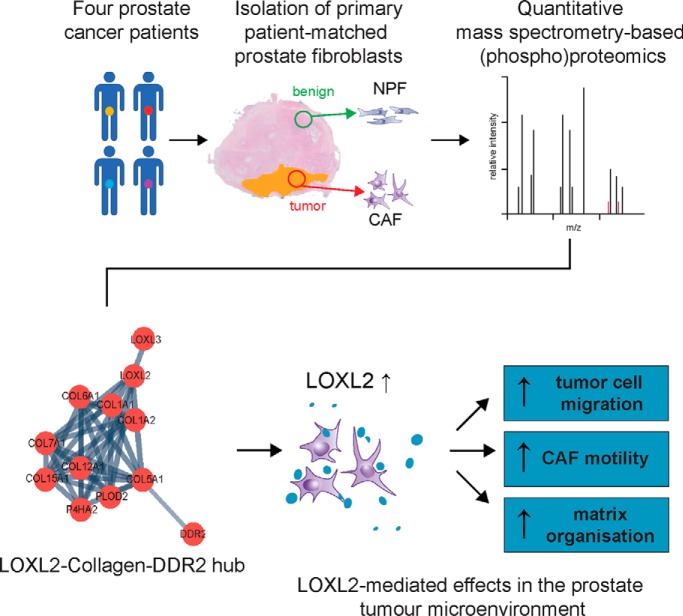

- Quantitative (phoshpo)proteome of primary cell cultures of patient-matched prostate CAF and NPF.
- Key CAF-associated proteins validated using orthogonal methodologies.
- LOXL2 inhibitors D-penicillamine and PXS-S2A impaired CAF migration and ECM alignment.
- Pre-treatment with LOXL2 inhibitors impaired migratory capacity of RWPE-2 cells in co-culture.

Although initially considered cell-autonomous, both the development and progression of solid tumors are now known to be markedly influenced by the stromal microenvironment (1). In the normal prostate, stromal cells tightly regulate epithelial development and differentiation (2, 3). However, during malignant progression, the transformed epithelium invades the surrounding stroma and activates the tumor-stromal niche (4). Alterations in the morphology and cellular composition of prostate cancer-associated stroma, as well as corresponding gene and protein expression profiles, correlate with tumor grade and prognosis which highlights an active role for tumor-stroma in disease progression (5–7).

The transition from normal to cancer-associated fibroblasts (CAF)^1^ is initiated in the early stages of tumorigenesis after which CAF co-evolve with tumor cells, influencing their pathogenesis and progression (8). CAF regulate multiple facets of the tumor microenvironment including growth factor and cytokine production, immune modulation, angiogenesis and extracellular matrix (ECM) deposition and remodeling (9). Primary cell cultures of patient-matched CAF and non-malignant prostate fibroblasts (NPF) can be derived following radical prostatectomy (RP) from malignant and benign regions of prostate tissue, respectively. Subsequent characterization has revealed that CAF have distinct alterations in their phenotype and function (10–13), with recent work indicating that this is encoded by differences in DNA methylation (14). Moreover, tissue recombination experiments identified that prostatic CAF retain the ability to promote tumorigenesis of “initiated” prostate epithelial cells *in vivo* (15, 16), and can enhance tumorigenic potential and invasiveness of prostate cancer cells *in vitro* (10, 17–21).

Several candidate mechanisms for intercellular communication between CAF and prostate epithelial cells have been identified. For example, several paracrine mediators exhibit enhanced expression in CAF, which include SFRP1, CXCL12, TGFβ1, HSP90, and FGF10 (22–26) whereas production of Hedgehog (Hh) ligands by the epithelial cells may initiate reciprocal signaling with CAF (27). In addition, gene set enrichment analysis of the differentially expressed genes between CAF and NPF revealed enrichment of functional categories for extracellular matrix, basal lamina and basement membrane (20). This is notable, because changes to the architecture and biophysical properties of the ECM influence tumor progression (28–31), and are prognostic biomarkers in multiple cancers (9). Yet, the reciprocal signaling that occurs between prostate epithelial cells, CAF and their ECM is still poorly understood. The prostate tumor microenvironment is likely to contain additional candidate biomarkers and therapeutic targets.

To address this knowledge gap, we have undertaken unbiased proteomic and phosphoproteomic profiling of patient-matched prostate CAF and NPF. This revealed important differences present in CAF, including changes in the ECM signaling network that collectively contribute to a protumorigenic microenvironment.

## EXPERIMENTAL PROCEDURES

### 

#### 

##### Isolation of Nonmalignant Prostate Fibroblasts (NPF) and Cancer-associated Fibroblasts (CAF) from Primary Prostate Tissue

Human prostate specimens were obtained following radical prostatectomy (RP) with the following human research ethics committee approvals: Cabrini Institute (03-14-04-08), Epworth HealthCare (34306 and 53611) and Monash University (2004/145).

RWPE-2 cells (American Type Culture Collection, Dublin, IRL) were maintained in keratinocyte serum free medium (KSF-M; Gibco) supplemented with 5 ng/ml epidermal growth factor (EGF; Gibco), 50 mg/ml bovine pituitary extract (BPE; Gibco), and 100 U/ml penicillin and 100 mg/ml streptomycin (P/S; Sigma-Aldrich, St. Louis, MS) at 37 °C, 5% CO_2_, with media changes every 2–3 days. CAF and NPF were directly isolated from patient tissue as previously described (11). Briefly, benign and tumor regions were identified and excised by a trained pathologist. Whole tissue was enzymatically digested to release cells into suspension and cultured in fibroblast media (RPMI 1640 (School of Biomedical Sciences, Media and Prep Services, Monash University)) supplemented with phenol red, 5% heat inactivated HyClone fetal bovine serum (HI-FBS; GE Healthcare), 1 nm testosterone (Sigma-Aldrich), 10 ng/ml basic fibroblast growth factor (bFGF; Merck Millipore, Burlington, MA) and P/S. Cells were maintained at 37 °C in 5% CO_2_, 5% O_2_ atmosphere, with media changes every 2–3 days. Matched CAF and NPF cell lines were established from cancer and benign tissue pieces respectively and verified via IHC at passage 4 to show homogenous expression of the fibroblast markers vimentin and α-smooth muscle actin and the absence of epithelial cytokeratins (20). Before use in this study, most (5/6) matched CAF and NPF pairs were validated using *in vivo* tissue recombination experiments whereby CAF, but not NPF, promoted tumorigenicity of initiated prostate epithelial cells as previously described (11). All patient information can be found in supplemental Table S1. Given that CAF and NPF are a primary, patient-derived line, early passage (3–8) CAF and NPF were used in this study unless otherwise stated.

##### Protein Preparation

Primary fibroblast cells were cultured in a 15 cm Petri dish until 80% confluent. Three biological replicates from each CAF or NPF line (Patients 1–4) were obtained from three separate passages. To harvest protein for mass spectrometry (MS) analysis, dishes were placed on a bed of ice and the cells were first washed twice with ice cold phosphate-buffered saline (PBS). Cells were lysed directly in the dish using 500 μl lysis buffer (8 m Urea, 20 mm HEPES, 2.5 mm sodium-pyrophosphate, 2.5 mm β-glycerol phosphate, 1 mm sodium orthovanadate, Roche protease inhibitors (1 tablet per 50 ml)). After scraping the lysed cells and transferring to a 1 ml Eppendorf tube, the samples were vortexed, sonicated and precipitated overnight with 4× volume of an ice-cold acetone solution (80% acetone, 10 mm NaCl). After this, samples were centrifuged at 3500 RPM for 15 mins. The supernatant was discarded, and the remaining cell pellet was stored at −80 °C until trypsin digestion. Total protein was measured using the Bicinchoninic acid protein assay (Bio-Rad, Hercules, CA). Protein extracts (100 μg) were denatured with 6 m urea in 25 mm ammonium bicarbonate before reduction with 5 mm TCEP at 37 °C for 1 h and alkylation with 32 mm iodoacetamide in the dark for 1 h. Alkylation was stopped by addition of 27 mm DTT. PNGase F (5000 U) was added to the sample mixture and incubated at 37 °C for 1 h. The samples were then diluted 1:10 with ammonium bicarbonate and digested with a 1:50 modified trypsin (Promega, Madison, WI) to protein weight at 37 °C for 18 h. Tryptic digests were acidified with 10% TFA to pH 2–3, desalted with a C18 column (Thermo Fisher Scientific, Waltham, MA) and eluted with 0.1% trifluoroacetic acid (TFA)/40% acetonitrile (ACN). Peptides were dried with a SpeedVac and re-suspended in 2% ACN/0.1% formic acid (FA) before mass spectrometry (MS) analysis.

##### Phosphopeptide Enrichment

For phosphopeptide enrichment, following desalting, 2 mg of peptides were enriched for 1 h with 2.5 mg of TiO_2_ (GL Science, Japan). Phosphopeptides were eluted with 150 μl of 0.3 m NH_4_OH, acidified with TFA to pH 2–3, and desalted immediately with a C18 column. Phosphopeptides were eluted manually with 50 μl of 0.1% FA/80% ACN and evaporated to dryness in a SpeedVac. The dried peptides were reconstituted in 2% ACN/0.5% FA.

##### Mass Spectrometry Analysis

Samples were analyzed on an UltiMate 3000 RSLC nano LC system (Thermo Fisher Scientific) coupled to an LTQ-Orbitrap mass spectrometer (LTQ-Orbitrap, Thermo Fisher Scientific). Peptides were loaded via an Acclaim PepMap 100 trap column (100 μm × 2 cm, nanoViper, C18, 5 μm, 100 Å, Thermo Fisher Scientific) and subsequent peptide separation was on an Acclaim PepMap RSLC analytical column (75 μm × 50 cm, nanoViper, C18, 2 μm, 100 Å, Thermo Fisher Scientific). For each liquid chromatography-tandem mass spectrometry (LC-MS/MS) analysis, 1 μg of peptides as measured by a nanodrop 1000 spectrophotometer (Thermo Fisher Scientific) was loaded on the pre-column with microliter pickup. Peptides were eluted using a 2 h linear gradient of 80% ACN/0.1% FA flowing at 250 nL/min using a mobile phase gradient of 2.5–42.5% ACN. The eluting peptides were interrogated with an Orbitrap mass spectrometer. The HRM DIA method consisted of a survey scan (MS1) at 35,000 resolution (automatic gain control target 5^e6^ and maximum injection time of 120 ms) from 400 to 1,220 *m*/*z* followed by tandem MS/MS scans (MS2) through 19 overlapping DIA windows increasing from 30 to 222 Da. MS/MS scans were acquired at 35,000 resolution (automatic gain control target 3^e6^ and auto for injection time). Stepped collision energy was 22.5%, 25%, 27.5%, and a 30 *m*/*z* isolation window. The spectra were recorded in profile type.

##### HRM-DIA Data Analysis

The DIA data were analyzed with Spectronaut 8, a mass spectrometer vendor-independent software from Biognosys. The default settings were used for the Spectronaut search. Retention time prediction type was set to dynamic indexed Retention Time (iRT; correction factor for window 1). Decoy generation was set to scrambled (no decoy limit). Interference correction on MS2 level was enabled. The false discovery rate (FDR) was set to 1% at peptide level. A peptide identification required at least 3 transitions in quantification. Quantification was based on the top 3 proteotypic peptides for each protein, normalized with the default settings, and exported as an excel file with Spectronaut 8 software (32). For generation of the spectral libraries, DDA measurements of each sample were performed. The DDA spectra were analyzed with the MaxQuant Version 1.5.2.8 analysis software using default settings. Enzyme specificity was set to Trypsin/P, minimal peptide length of 6, and up to 3 missed cleavages were allowed. Search criteria included carbamidomethylation of cysteine as a fixed modification; oxidation of methionine; acetyl (protein N terminus); and phosphorylation of serine, threonine, and tyrosine as variable modifications. The mass tolerance for the precursor was 4.5 ppm and for the fragment ions was 20 ppm. The DDA files were searched against the human UniProt fasta database (v2015–08, 20,210 entries) and the Biognosys HRM calibration peptides. The identifications were filtered to satisfy FDR of 1% on peptide and protein level. The spectral library was generated in Spectronaut and normalized to iRT peptides.

##### Mass Spectrometry Statistical Analysis

Log_2_ intensities of the peptides were summarized across all the samples in a linear mixed model implemented in the R package MSstats (33) for pairwise comparison for each protein or phosphopeptide. The *p* values were adjusted for multiple testing using the Benjamini-Horchberg method (34) with an FDR <0.05 and a fold change of >1.5 was required for differential expression.

##### Functional Annotation Analysis

Functional annotation of the CAF and NPF proteomes and phosphoproteomes was conducted using database for annotation, visualization, and integrated discovery (DAVID) software (35). Overrepresented functional categories among proteins enriched in each sample population was relative to a background of all identified proteins. Criteria for reported functional enrichment required a fold enrichment >1.5, FDR <0.05, and adjusted *p* value <0.05. Experimentally verified and published protein-protein interactions from several resources including STRING (36) and the Matrisome Project (37) were assessed.

##### Western Blot Analysis

Primary fibroblast cells were cultured in a 15 cm Petri dish until 80% confluent. To harvest protein for Western blotting, dishes were placed on a bed of ice and the cells were first washed twice with ice-cold PBS. Cells were lysed directly in the dish using 200 μl of radioimmune precipitation assay buffer (RIPA; Millipore) with protease and phosphatase inhibitors (1 mm phenylmethylsulfonyl fluoride, 10 μg/ml aprotinin, 10 μg/ml leupeptin, 1 mm sodium fluoride and 1 mm sodium orthovanadate). After scraping the lysed cells and transferring to a 1 ml Eppendorf tube, the samples were briefly vortexed. After this, the samples were centrifuged at 10,000 RPM for 10 min at 4 °C. Protein concentrations in the resulting supernatant were determined using a reducing agent and detergent compatible (RC DC) protein assay kit (Bio-Rad). Equal amounts of protein (between 10 and 30 μg per lane) were separated by SDS-PAGE on an 8 or 10% gel and transferred to PVDF membranes (Millipore, Minneapolis, MN). Antibodies detecting LOXL2 (#55470; Abcam, Cambridge, UK; 1:2000), DDR2 (#AF2538; R&D Systems, Minneapolis, MN; 1:4000), FAK (#610088; BD Biosciences, Franklin Lakes, NJ; 1:1000), phospho-FAK 925 (#3284; Cell Signaling Technology, Danvers, MA; 1:1000) and α-tubulin (#T5168; Sigma-Aldrich; 1:5000) were incubated overnight at the indicated dilutions at 4 °C. β-actin (#a5441; Sigma-Aldrich; 1:200,000) was incubated for 30 min at 4 °C. After washing, the blots were incubated with either polyclonal goat α-rabbit immunoglobulins/HRP, polyclonal goat α-mouse immunoglobulins/HRP or polyclonal rabbit α-goat immunoglobulins/HRP (all 1:10,000, Dako, Santa Clara, CA) in 5% (w/v) skimmed milk powder + 0.05% (v/v) Tween 20 (TBST) blocking solution for 1 h at room temperature. Detection was by enhanced chemiluminescence and utilized ChemiDoc Touch Imaging system (Bio-Rad). Signals were quantitated using Image Lab (Bio-Rad) or ImageJ (NIH). Statistical analyses were performed using GraphPad Prism 7 software (GraphPad Software Inc.). Pooled densitometry values (*n* = 6) were subjected to a paired, one-tailed Wilcoxon test (*p* value <0.05) to determine statistical significance. Uncropped western blots are shown in supplemental Figs. S4–S5.

##### Flow Cytometry

Two million patient-matched CAF or NPF were co-stained with FITC mouse anti-human CD90 (clone 5E10; BD Biosciences, San Diego, CA) and PE anti-human CD166 (clone 3A6; Biolegend) for 20 min on ice in FACS Buffer (PBS containing 10% HI-FBS and 5 mm EDTA). Cells were washed with PBS and re-suspended in 200 μl of FACS buffer containing 1 μg/ml propidium iodide (PI) to label dead cells. 3 × 10^5^ live cell events were collected on the LSR II flow cytometer (BD Biosciences) and analyzed using FlowJo software v10 (BD Biosciences). Statistical significance was determined using a Mann-Whitney *U* test (*p* value <0.05).

##### The Cancer Genome Atlas Data

Gene expression and clinical information were derived from the National Cancer Institute GDC Data Portal (https://portal.gdc.cancer.gov/projects/TCGA-PRAD), containing 492 patients RNA-seq data. Briefly, patients were stratified to high and low LOXL2 gene expression using a cut-off of 0.6. Their disease-free survival (DFS) was plotted using the Kaplan-Meier curve and differences in DFS were evaluated using the log-rank statistical test (*p* value <0.05).

##### LOX/LOXL2 Enzyme Activity Assay

LOX/LOXL2 function was measured as previously described (38). The conditioned media from CAF/NPF from Patients 4, 6, and 7 or media control was collected following 72 h culture in fibroblast media (phenol red free and 1% HI-FBS), and concentrated by size filtration (MWCO 10kDa). LOX/LOXL2 enzymatic activity was assessed using the standard Amplex Red, based on the production of H_2_O_2_ and the substrate putrescine as described previously (39, 40). The relative fluorescence units (RFU) were read every 2.5 min for 30 min at 37 °C, excitation 565 nm and emission 590 (Optima, BMG labtech, Ortenberg, DEU) and expressed in RFU/30 min. 100 μm of the LOX/LOXL inhibitor β-aminopropionitrile (BAPN) and 1 μm of the LOXL2 specific inhibitor PXS-S2A (Pharmaxis, New South Wales, Australia) were added to reactions to demonstrate specificity of fluorescent signal (40). The difference between the signal obtained in the presence or absence of BAPN or PXS-S2A inhibitors in CAF/NPF samples was considered specific for LOX/LOXLs or LOXL2 activity respectively. Statistical significance was determined using a one-way ANOVA with Sidak's post-hoc multiple comparisons test (*p* value <0.05).

##### ECM Orientation Analysis with Drug Treatment

CAF cultures were treated with either 10 and 50 μm d-penicillamine (DPEN; Sigma-Aldrich, Australia) or 10 and 100 nm PXS-S2A every 2–3 days until confluent. MilliQ H_2_O or DMSO were used as vehicle controls for DPEN and PXS-S2A, respectively. The orientation of ECM fibers was analyzed using ImageJ (NIH) plugin OrientationJ as previously described (41, 42). Briefly, a representation of the angles of the ECM fibers was characterized by hue-saturation-brightness (HSB) color coded images, where the different colors relate to different absolute angles of orientation. The distribution of orientation angles was assessed by analyzing the orientation and isotropic properties of individual pixels that together made up the ECM fibers. A cubic spline gradient interpolation with a Gaussian window of σ = 2 was applied, which gave quantitative data for the distribution and frequency of angles from −90° to 90°. After normalization of the orientation peak distributions, plots were subjected to a Kruskal-Wallis test with Dunn's post-hoc multiple comparisons test (*p* value <0.05) to determine statistical significance.

##### CAF Wound Healing Assay with Drug Treatment

Mitomycin C-treated cells were cultured in 2-well culture inserts (Ibidi) in fibroblast media until 80% confluent. A manual line (Gap ∼500 μm) was established in CAF cultures by removal of the plastic insert. CAF were then assessed for their ability to close the physical gap in the presence or absence of 10 μm and 50 μm LOXL2 inhibitor d-penicillamine (vehicle control: MilliQ H_2_O) or 10 nm and100 nm PXS-S2A (vehicle control: DMSO). Images were acquired every hour for 24 h using a Leica AF600LX Live cell microscope (Wetzlar, Germany). Images were processed using ImageJ software and a border was manually applied to the gap area from each condition. The percentage gap closure at 24 h was normalized to vehicle control. Statistical significance was determined using a one-way ANOVA with Dunnett's post-hoc multiple comparisons test (*p* value <0.05).

##### CAF/RWPE-2 Migration Assay with Drug Treatment

Patient CAF were seeded into 24 well tissue culture plates and cultured in fibroblast media over ∼5 d to establish matrix (or until 80% confluent). Cultures were pre-treated with 10 μm and 50 μm LOXL2 inhibitor d-penicillamine (vehicle control: MilliQ H_2_O) or 10 nm and 100 nm PXS-S2A (vehicle control: DMSO) for 24 h, before drug washout with PBS and replacement with fresh drug-free fibroblast media. Tumorigenic RWPE-2 cells (American Type Culture Collection) were labeled for 30 min with CellTracker (CT) green (Invitrogen, Carlsbad, CA) before addition to the pretreated CAF cultures and imaged immediately. Images were acquired every 20 min for 12 h with a Leica AF600LX Live cell microscope (Wetzlar, Germany). Videos were processed to analyze RWPE-2 migration using Imaris software (Bitplane AG, Switzerland). Statistical significance was determined using a one-way ANOVA with Dunnett's post-hoc multiple comparisons test (*p* value <0.05).

##### Immunofluorescence

CT-green labeled RWPE-2 and CAF co-cultures were fixed in 4% paraformaldehyde (Sigma-Aldrich) for 15 min at room temperature, washed twice with PBS and then permeabilized for 10 min with 0.1% Triton X-100 (BDH) in PBS. Fixed cells were blocked for 10 min using 1% bovine serum albumin (BSA; Sigma-Aldrich) in PBS. To visualize the extracellular matrix, cultures were stained with mouse anti-human fibronectin (clone HFN 7.1; DSHB) and/or rabbit polyclonal anti-collagen I (ab34710; Abcam) for 1 h at room temperature. Secondary labeling was performed with anti-mouse Alexa Fluor 647 and goat Anti-Rabbit IgG Alexa Fluor 555 (Cell Signaling Technology), respectively, for 30 mins at room temperature. F-actin was visualized with Rhodamine Phalloidin (Thermo Fisher Scientific). Confocal images were acquired on a Nikon C1 Inverted Eclipse 90i confocal microscope equipped with 20× objective lens (Nikon) using NIS Elements Software (Nikon).

##### Experimental Design and Statistical Rationale

Three biological replicates from four patient-derived matched CAF and NPF cell lines (Patients 1–4) were processed in the initial whole proteome analysis; one patient sample had only two biological replicates because of sample unavailability. For phosphoproteome analysis, a pool of three biological replicates for each patient-derived matched CAF and NPF cell line (Patients 1–4) was processed to obtain 2 mg of peptides before TiO_2_ enrichment. Data was analyzed both in data-dependent acquisition (DDA) mode (to generate a spectral library) and in data-independent acquisition (DIA) mode. Protein quantification from the DIA analysis mandated at least three transitions of the top three proteotypic peptide. Data was normalized with the Spectronaut 8 software and MSstats (version 3.2.2) was implemented to fit an appropriate linear-mixed model for pairwise comparison.

For functional validation, the four patient-derived matched CAF and NPF cell lines analyzed in the whole proteome and phosphoproteome analysis were utilized (Patient 1–4). In addition, a further two validation lines (Patient 5 and 6) were used where possible. Because of the finite lifespan of primary cells it was not possible to perform all assays on every CAF/NPF pair. Validation of proteins CD90 and CD166 (via flow cytometry) and DDR2, LOXL2, FAK, and pFAK925 expression (via Western blotting) were assessed in Patients 1–6; LOX/LOXL2 enzymatic function (Patients 4–6); CAF wound healing assay and the CAF/RWPE-2 co-culture migration assay in the presence of DPEN (Patients 4–6) or PXS-S2A (Patients 4 and 6). Statistical analysis for each individual assay is reported in the specific methods section.

## RESULTS

### 

#### 

##### Global (phospho)proteomic Analysis of Matched NPF and CAF

**Fig. 1.**
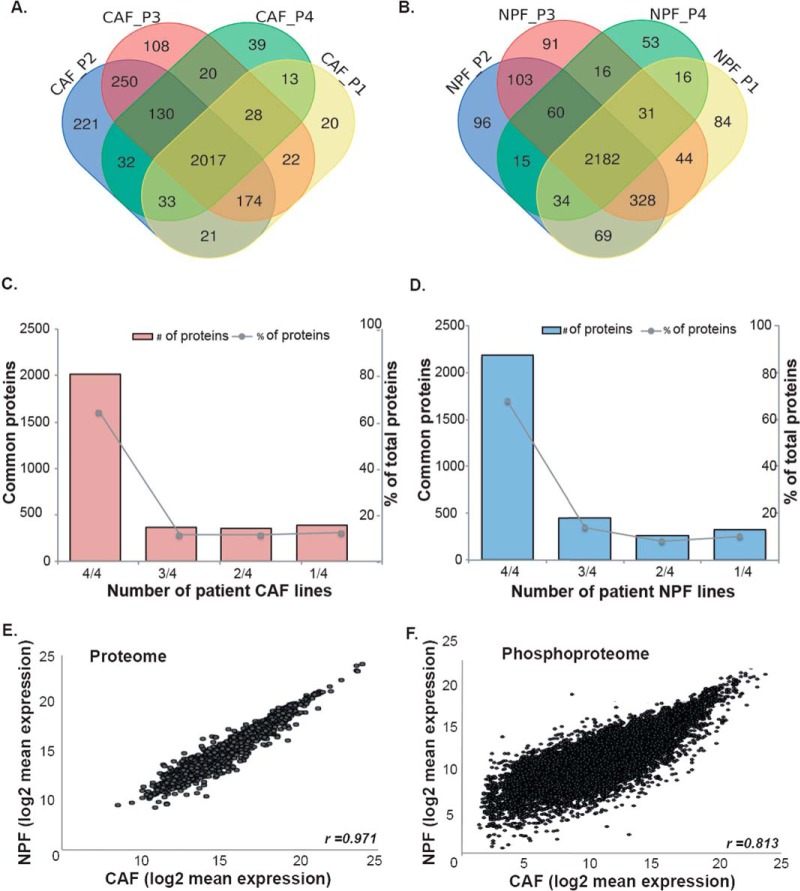
**Comparison of the proteomes and phosphoproteomes of prostate CAF and NPF.** Venn diagrams of overlapping proteins identified in the spectral library of the (*A*) CAF proteome and (*B*) NPF proteome. Bar graphs of common proteins (*left axis*) and the percentage of total identified proteins (*right axis*) in each number of patient cell lines in the spectral library of (*C*) CAF proteome and (*D*) NPF proteome. Scatter plots comparing the average log_2_ expression of (*E*) individual proteins and (*F*) phosphopeptides in matched CAF *versus* NPF patient-derived lines (*n* = 4).

To investigate the functional differences between prostate CAF and NPF, we characterized their respective proteomes and phosphoproteomes by LC-MS/MS with a hyper-reaction monitoring data-independent acquisition (HRM-DIA) workflow. We analyzed four patient-matched NPF/CAF pairs from moderate to high grade prostate cancer (P1-P4; supplemental Table S1). First, a DDA workflow was used to generate a spectral library. This identified 4,586 proteins across all samples (supplemental Table S2). Most proteins were consistently detected across patients, with 82% of proteins in NPF and 76% of proteins in CAF detected in cells derived from three or more patients (Fig. 1*A*–1*D*). Consequently, although there is detectable inter-patient heterogeneity among these cells, there are also significant similarities. To interrogate inter-patient heterogeneity, functional pathway analysis was used to compare proteins identified in only one or two patients compared with proteins identified in at least three cell lines. There was a significant enrichment of pathways associated with mitochondrial translation and poly (A) RNA binding among the proteins that were only detected in a minority of CAF. No specialized pathways were enriched in proteins detected in the minority of NPF lines. Proteins detected in the majority of CAF and NPF lines were enriched for extracellular exosome, poly (A) RNA binding, focal and cell-cell adhesion pathways (supplemental Table S3).

We then used a comprehensive HRM-DIA workflow based on the generated spectral library to detect and quantify 4075 proteins that are present in all NPF and CAF (supplemental Table S4). Quantitative data for these proteins are presented in supplemental Table S5. The coefficients of variation of protein expression were found to be less than 10.5% across biological replicates in patient samples for both quantification approaches (DDA and DIA), except for the CAF replicates from Patient 1. This demonstrates low variability among biological replicates for most of the CAF/NPF lines. The proteomic profile of CAF and NPF was highly similar for the 4075 proteins detected by the DIA workflow (*r* = 0.971; Fig. 1*E*). In addition, we used a TiO_2_-enrichment workflow to identify 12,209 phosphorylated peptides present in all NPF and CAF lines, corresponding to 3032 proteins (supplemental Table S6 and S7). Of the phosphorylated proteins identified by this approach, 1409 were also identified in the proteome analysis. The correlation for the phosphoproteomic profile between CAF and NPF was strong but slightly less than that observed for the whole proteome (*r* = 0.813; Fig. 1*F*).

##### Identifying Differentially Expressed Proteins Capable of Discriminating CAF and NPF Samples Within An Interpatient Proteome Landscape

**Fig. 2.**
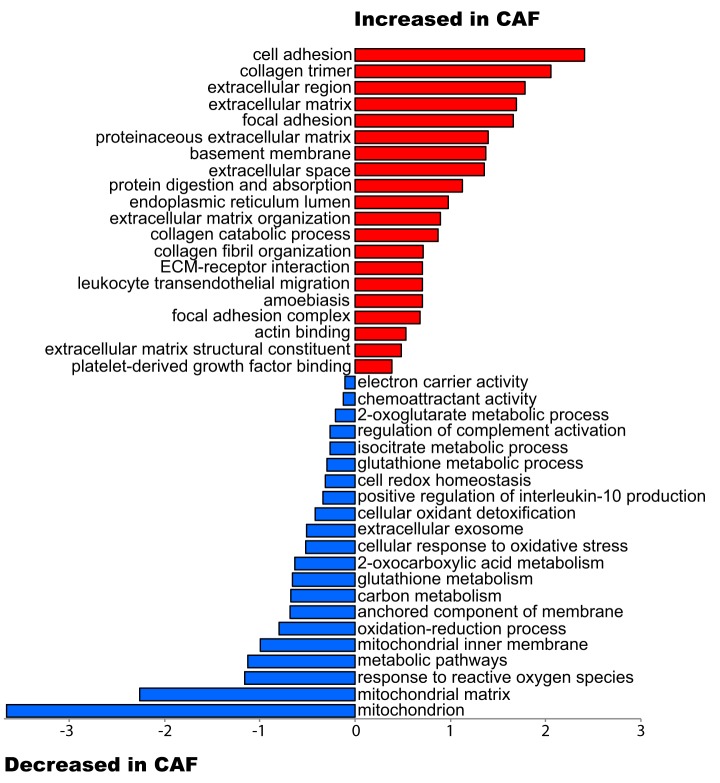
**Functional analysis of differentially abundant proteins in prostate CAF compared with NPF.** The plot shows functional categories that are over-represented relative to all identified proteins using a permutation-based false discovery rate (FDR) analysis.

**Fig. 3.**
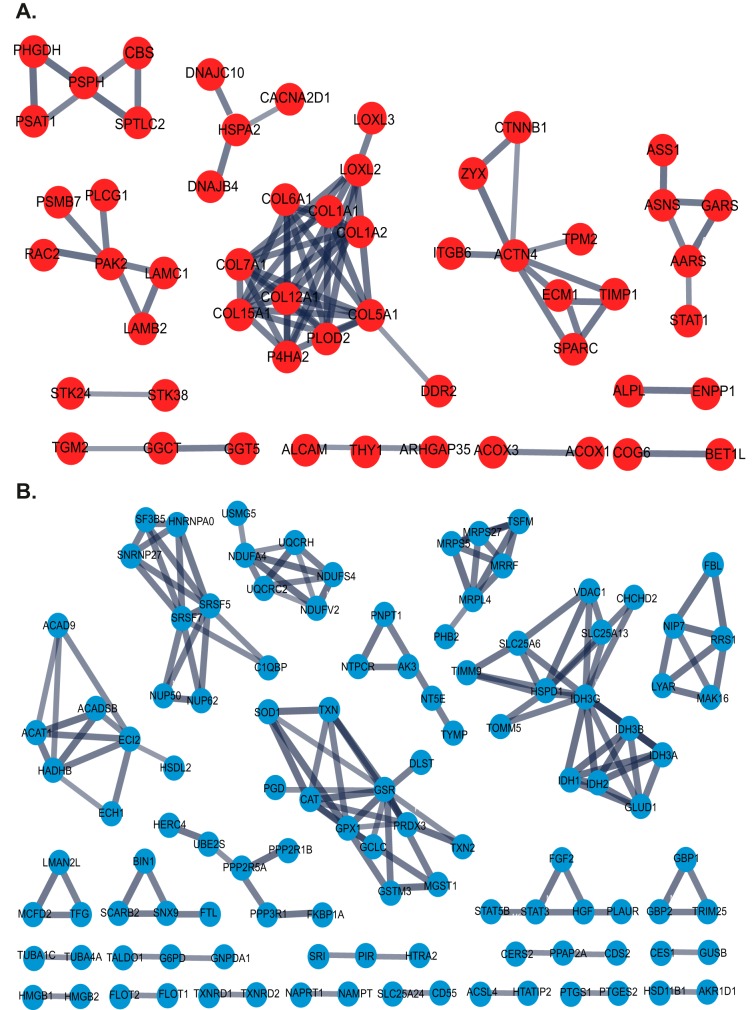
**STRING network analysis of differentially abundant proteins.** Networks formed by proteins with (*A*) increased abundance and (*B*) decreased abundance in CAF *versus* NPF.

Differential expression analysis of patient-matched CAF and NPF pairs identified 363 differentially-expressed proteins between CAF and NPF (raw *p* value ≤0.02 and fold change >1.25) (supplemental Table S8). Functional pathway analysis with DAVID revealed CAF samples were enriched for proteins involved in cell adhesion and the extracellular matrix, and NPF samples were enriched for proteins involved in the mitochondrion, the oxidation-reduction process and metabolic pathways (Fig. 2). Network analysis using the STRING database was performed on proteins with significantly increased abundance in CAF and highlighted a prominent protein-protein interaction hub involved in collagen expression, regulation and signaling (Fig. 3*A*). Specifically, this hub contained multiple collagens, including the fibrillar types COL1A1/2 and COL5A1, LOXL2/LOXL3, which are copper-dependent amine oxidases that promote collagen crosslinking (43), and the receptor tyrosine kinase DDR2 that acts as a receptor for fibrillar collagens (44) (Fig. 3*A*). In addition, several non-fibrillar collagens were more abundant including COL6A1, COL7A1, COL12A1, and COL15A1. We note that collagens function as members of heterotrimeric assemblies, and for Type I collagen, all members of the complex (*i.e.* COL1A1 and COL1A2) met our threshold for increased expression in CAF. In the case of other collagen types, such as Type V and VI, certain members met the threshold (*e.g.* COL5A1 and COL6A1) whereas their partners either showed a trend that did not meet the cut-off (*e.g.* COL5A2, COL6A2) or did not change (*e.g.* COL6A3). Two enzymes involved in collagen modification and folding (PLOD2 and P4HA2) (45, 46) were also present. These data are consistent with enrichment for the functional terms “cell adhesion,” “collagen trimer,” and “extracellular matrix” in CAF (Fig. 2). Also of interest were two hubs involved in cytoskeletal organization centered on the actin binding protein ACTN4 and the serine/threonine kinase PAK2 (Fig. 3*A*). Network analysis of proteins with decreased expression in CAF identified several hubs involved in cellular metabolism and reprogramming and mitochondrial function including IDH3A (47), IDH3G, UQCRH, MRPL4 and ACADSB, as well as cellular redox regulation (GSR) (Fig. 3*B*), explaining enrichment for the functional terms “mitochondrion,” “metabolic pathways,” and “oxidation-reduction process” in the NPF group (Fig. 2).

**Fig. 4.**
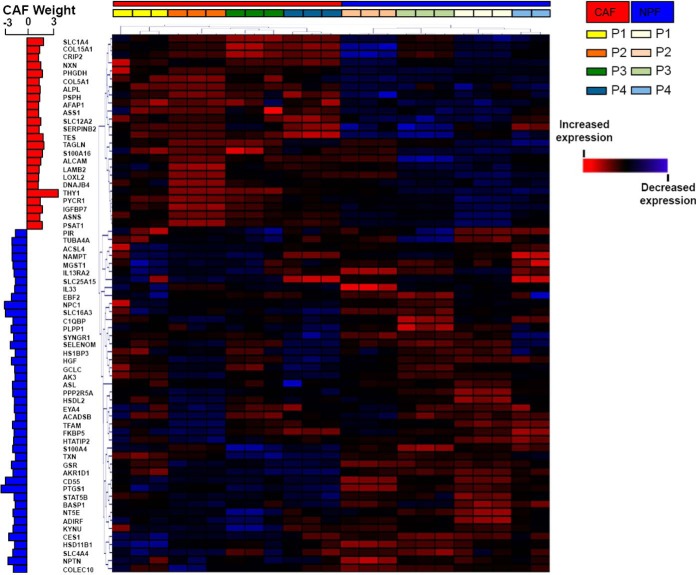
**Unsupervised hierarchical clustering of samples using proteins enriched in prostate CAF or NPF.** Clustering was undertaken using differentially abundant proteins selected using a stringent cut-off of adjusted *p* value <0.05 and fold change >1.5. The positive or negative CAF weight indicates the degree of significance relative to NPF.

We refined the list of proteins using a more stringent criterion (adjusted *p* value <0.05 and fold change >1.5) and identified 67 proteins that were differentially expressed between patient-matched CAF and NPF (supplemental Table S8). Unbiased hierarchical clustering of these proteins confirmed complete segregation of CAF and NPF (Fig. 4). The two proteins with the highest increase in expression in CAF were the THY1 cell surface antigen (adjusted *p* value 2.57E^−04^) and Transgelin (TAGLN; adjusted *p* value 1.14E^−02^). Other ECM proteins, such as Laminin B2 along with its binding partner γ1 (LAMC1) and Lysyl Oxidase 2 (LOXL2), were also increased in CAF compared with NPF. Another Laminin B2 interactor, Laminin subunit α2 (LAMA2) was increased in CAF compared with NPF but did not meet our cut-off threshold.

##### Identifying Phosphosites that Exhibit Contrasting Abundance in CAF and NPF

To identify differences in phosphorylation-based signaling between CAF and NPF, we used TiO_2_ enrichment coupled with mass spectrometry analysis (supplemental Table S6). We identified 161 phosphopeptides that differed in abundance between CAF and NPF (raw *p* value <0.01, FC >1.5). These phosphopeptides mapped to 138 proteins (supplemental Table S9). We did detect some ECM-affiliated phosphopeptides (45/12,209 phosphopeptides). The most abundant from this limited list were p2384-FN1, p45-MGP, p192 p201-IGFBP3, p72-TNC, p52 p53 p116 p118 p132 p133 p267-MFAP1, p258-COL16A1, p70-CRELD2, and p942 p943-COL20A1. However, the majority of phosphopeptides that significantly differed in abundance between CAF and NPFs were not ECM-related (supplemental Tables S6 and S9).

**Fig. 5.**
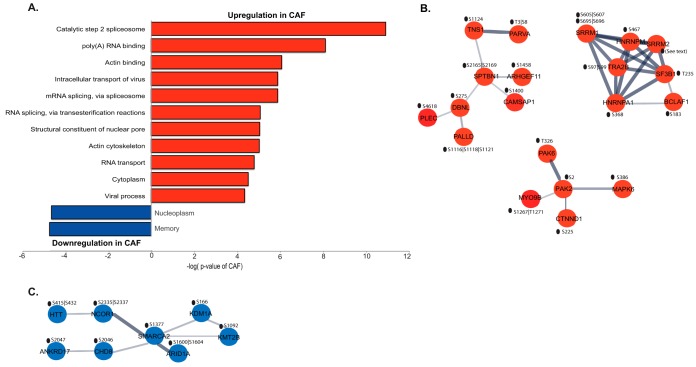
**Characterization of proteins with differential phosphorylation between CAF and NPF.** *A*, Functional analysis of differentially-phosphorylated proteins. Over-represented functional categories are relative to all identified proteins using a permutation-based false discovery rate (FDR) analysis. STRING network analysis for proteins with (*B*) increased and (*C*) decreased phosphorylation in CAF *versus* NPF. The differentially phosphorylated sites are shown.

Functional analysis of proteins exhibiting increased phosphosite abundance in CAF showed enrichment for categories associated with RNA metabolism and the actin cytoskeleton (Fig. 5*A*). Network analysis using STRING revealed prominent interaction hubs involving mRNA processing (TRA2B) and the actin cytoskeleton (SPTBN1) (Fig. 5*B*). Interestingly, STRING analysis for proteins exhibiting decreased phosphosite abundance in CAF revealed an interaction hub associated with chromatin remodeling and transcriptional regulation (SMARCA2) (Fig. 5*C*). supplemental Fig. S1 contains the complete interaction network of differentially phosphorylated proteins. These data complement the proteomic analysis, confirming the actin cytoskeleton as a subcellular compartment with significant differences in composition and regulation between CAF and NPF. Further, regulatory mechanisms governing gene expression were identified that likely underpin functional differences between the two cell types.

##### Validation of CAF-associated Proteins

**Fig. 6.**
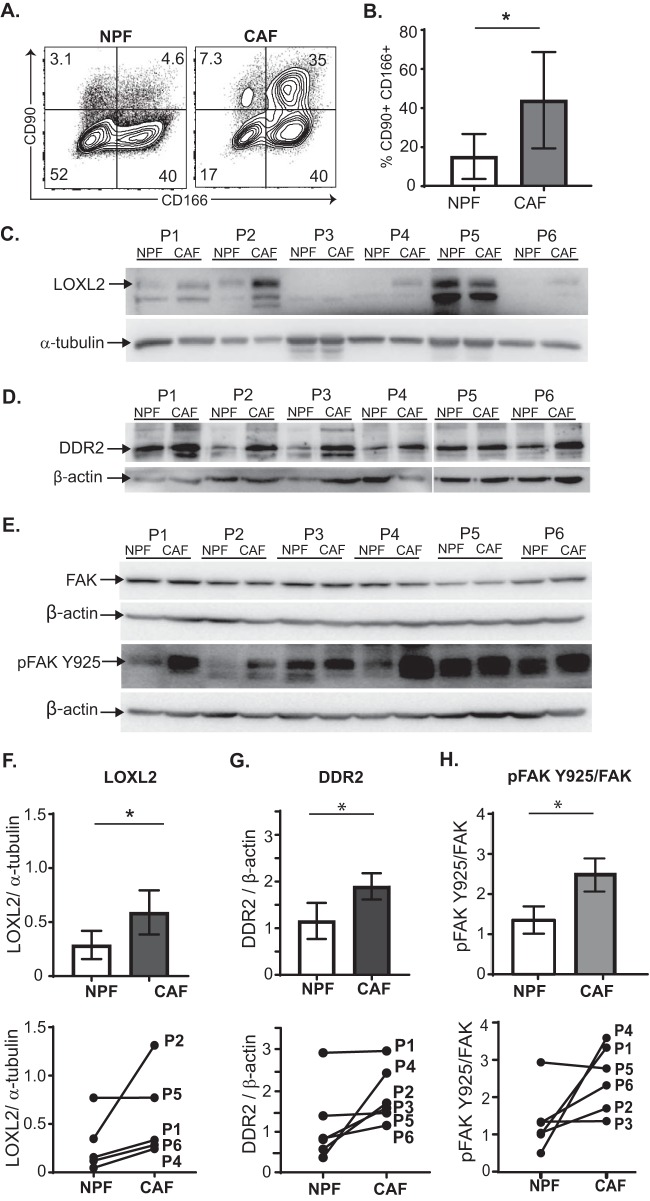
**Protein validation in patient-matched prostate CAF and NPF.** *A*, Representative flow plots show surface expression of CD90 and CD166 antigen on NPF and CAF (gated on propidium iodide-negative live cells). *B*, Graph shows the mean percentage (± S.D.) of CD90 and CD166 double-positive cells in NPF and CAF from Patient 1–6 (P1–6; supplemental Fig. S2). *p value <0.05 *C*, Western blots show LOXL2 protein expression and α-tubulin loading control in patient-matched NPF and CAF (P1–6); 20 μg of protein was loaded per lane (10 μg was loaded for P2). LOXL2 blots on biological replicate samples are shown in supplemental Fig. S3*A*–S3*B*. *D*, Western blots show DDR2 and β-actin loading control in patient-matched NPF and CAF (P1–6); 20 μg of protein was loaded per lane. *E*, Western blots of phospho-FAK Y925 and total FAK levels; β-actin was used a loading control; 15 μg of protein was loaded per lane. Densitometry of biological replicate shown in supplemental Fig. S3*C*. Quantification of blots by densitometry shows the average fold-change and patient-matched expression levels for; *F*, LOXL2, (*G*) DDR2 and (*H*) pFAK925/total FAK levels in CAF and NPF from Patients 1–6. FAK and pFAK protein expression was first quantified relative to their respective β-actin loading controls. Bars represent the mean ± S.E. (*n* = 6 patients). *p value <0.05 compared with NPF.

Where possible, protein validation was performed on the four patient-matched NPF/CAF pairs (Patient 1–4) used in the original (phospho) proteomic analysis, as well as two additional validation patients (Patient 5 and 6). Elevated THY1 (CD90) expression has previously been reported within prostate tumor stroma, but ALCAM (CD166) has not been previously investigated (10, 48). Flow cytometric analysis confirmed a significant increase in the expression of both CD90 and CD166 surface antigens on CAF *versus* NPF in the majority of patients (Fig. 6*A*–6*B* and supplemental Fig. S2).

We also verified components of the LOXL2/collagen/DDR2 signaling axis, highlighted by network analysis, using Western blot analysis of Patients 1–6 (P1–6). The expression of both LOXL2 (Fig. 6*C*, 6*F* and supplemental Fig. S3*A*–S3*B*) and DDR2 (Fig. 6*D* and 6*G*) was significantly increased in primary CAF lines compared with NPF. Previous work has shown that LOXL2 indirectly activates FAK/SRC signaling via enhanced ECM stiffness (49, 50). In addition, phosphorylation at Y925 is required for FAK-dependent cell protrusion, migration and invasion (50–52). Western blot analysis showed a similar level of total FAK protein between CAF and NPF, however increased phosphorylation at Y925 relative to total FAK was observed in CAF (Fig. 6*E*, 6*H* and supplemental Fig. S3*C*). Collectively, these data validate the upregulation of LOXL2 and DDR2 in prostate CAF and further reveal increased phosphorylation of FAK at Y925.

##### LOXL2 Mediates Autocrine and Paracrine Signaling in the Prostate Cancer Tumor Microenvironment

LOXL2 expression is up-regulated in prostate cancer compared with normal tissue (53) and is associated with poor prognosis in a variety of cancers (54). To demonstrate clinical relevance of LOXL2 we interrogated the prostate cancer RNA-seq data set (*n* = 492) of The Cancer Genome Atlas (TCGA) for LOXL2 gene expression levels in patients with localized prostate cancer. The results demonstrate that patients expressing higher levels of LOXL2 had a significant worsening of disease-free survival (DFS; hazard ratio 1.51; 95% confidence intervals 0.99–2.29; supplemental Fig. S6). Further, LOXL2 can be targeted by either small molecule or antibody-based approaches, highlighting the potential for rapid research translation (40). These findings and developments led us to interrogate the functional role of prostate CAF-dependent LOXL2 production.

**Fig. 7.**
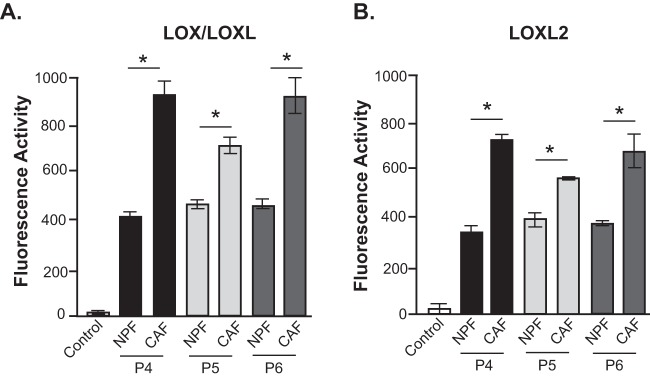
**LOX/LOXL2 enzymatic function in prostate CAF.** (*A*) LOX/LOXL and (*B*) LOXL2 enzymatic activity was measured in the conditioned media from patient-matched NPF and CAF (P4–6). Specific LOX/LOXL and LOXL2 enzymatic function was determined via the addition of BAPN (100 μm) and PXS-S2A (1 μm) inhibitors respectively. Bars represent mean ± S.E. (*n* = 3). *p value <0.05 compared with NPF.

**Fig. 8.**
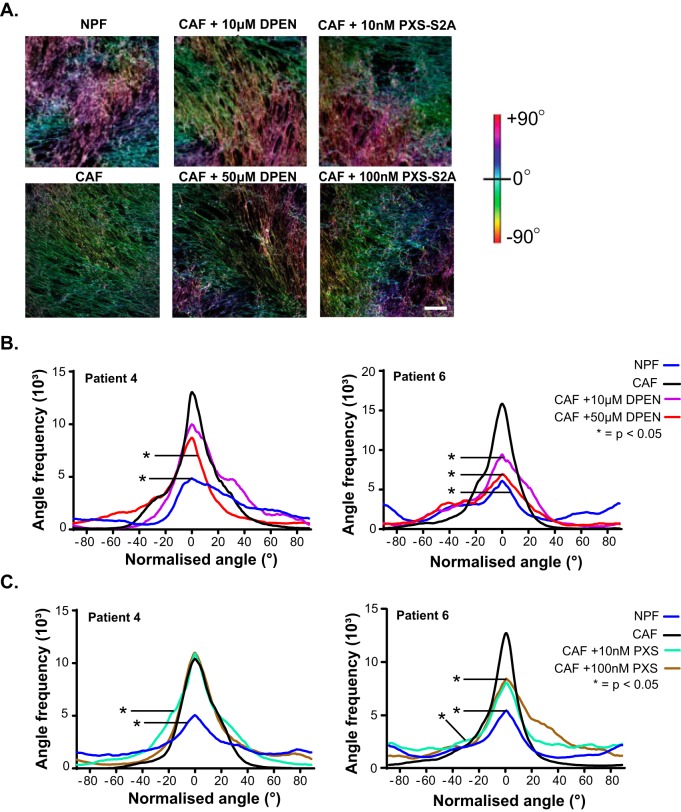
**LOX/LOXL2 inhibition abrogates extracellular matrix orientation in prostate CAF.** *A*, Representative images of fibronectin staining show ECM fiber alignment for cell-derived matrices produced by NPF and CAF or CAF treated with the LOX/LOXL2 inhibitor DPEN or selective LOXL2 inhibitor PXS-S2A. Images were processed and color-coded to represent the degree of fiber orientation distribution within each sample; scale bar = 50 μm. Quantification of fiber alignment for NPF and CAF treated with *B*, DPEN or (*C*) PXS-S2A from Patient 4 and 6. Lines represent mean values (*n* = 3). *p value <0.05 compared with CAF. Raw images are shown in supplemental Fig. S7.

Functional assessment of LOX/LOXL enzymatic activity (38) in conditioned media from patient matched prostate NPF/CAF pairs revealed increased secretion of active LOX/LOXL family members in prostate CAF (Fig. 7*A*). Notably, most of the enzyme activity was derived specifically from LOXL2 (Fig. 7*B*). Primary mesenchymal cells are often sensitive to transfection protocols, and indeed, our attempts to use siRNA transfection to interrogate the functional role of LOXL2 resulted in major cell death. Therefore, to assess the role of LOX/LOXL2 in ECM organization, we treated CAF and NPF *in vitro* cultures with the LOX/LOXL2 inhibitor d-penicillamine (DPEN) (55) or the small molecule inhibitor, PXS-S2A (40). PXS-S2A is a potent and highly selective LOXL2 inhibitor that does not exhibit any auxiliary pharmacology in standard profiling assays and displays greater than 500-fold selectivity for LOXL2 over other related human amine oxidases (40). Representative images demonstrate the disorganized ECM of NPF cell derived matrices based on analysis of fibronectin (Fig. 8*A*) and collagen I staining (supplemental Fig. S7), compared with the highly-aligned ECM fibers produced by prostate CAF. Treatment with DPEN abrogated ECM fiber alignment in CAF at 10 μm, and to a greater extent, 50 μm, whereas PXS-S2A abrogated CAF ECM at 10 and 100 nm concentrations. ECM fiber alignment was quantified in primary NPF/CAF cultures from Patients 4 and 6 and plotted to show the normalized frequency of fiber orientation. CAF fibers were significantly more orientated than matched NPF samples in both patients. Following DPEN and PXS-S2A treatment of CAF for 24 h, the ECM orientation of Patient 4 was perturbed when treated with 50 μm DPEN or 10 nm PXS-S2A, whereas Patient 6 CAF responded at both 10 and 50 μm DPEN and 10 and 100 nm PXS-S2A (Fig. 8*B*–8*C*, supplemental Fig. S7).

**Fig. 9.**
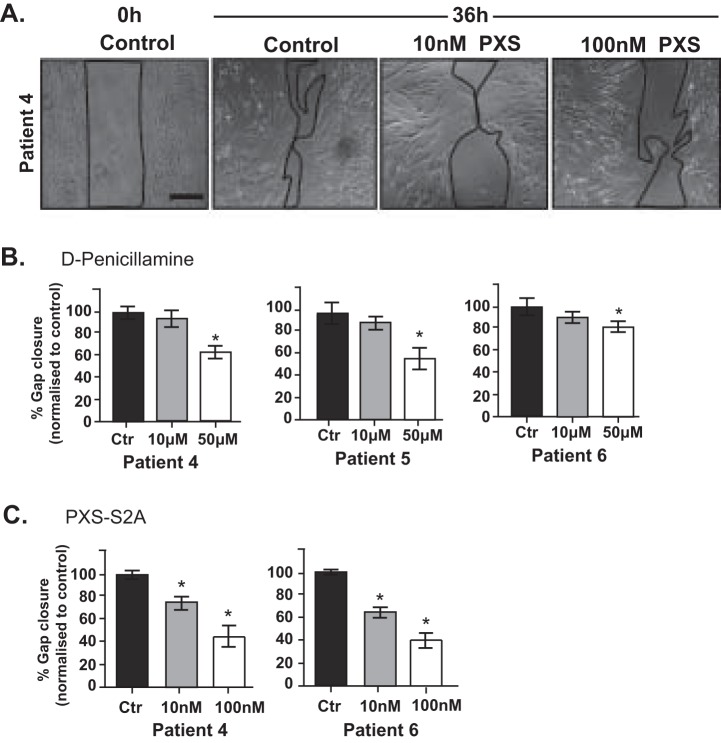
**LOX/LOXL2 inhibition impairs CAF migration.** *A*, Representative images of CAF cultures in the wound healing/scratch assay exposed to vehicle control (control; ctr) or PXS-S2A treatment for 36 h. Scale bar = 100 μm. The percentage gap closure was normalized and presented relative to control CAF (Ctr) at 36 h in the presence of (*B*) the LOXL/LOXL2 inhibitor d-Penicillamine (Patient 4–6) or (*C*) the selective LOXL2 inhibitor PXS-S2A (Patient 4 and 6). Graph bars represent mean ± S.E. (*n* = 6). *p value <0.05 relative to control CAF.

**Fig. 10.**
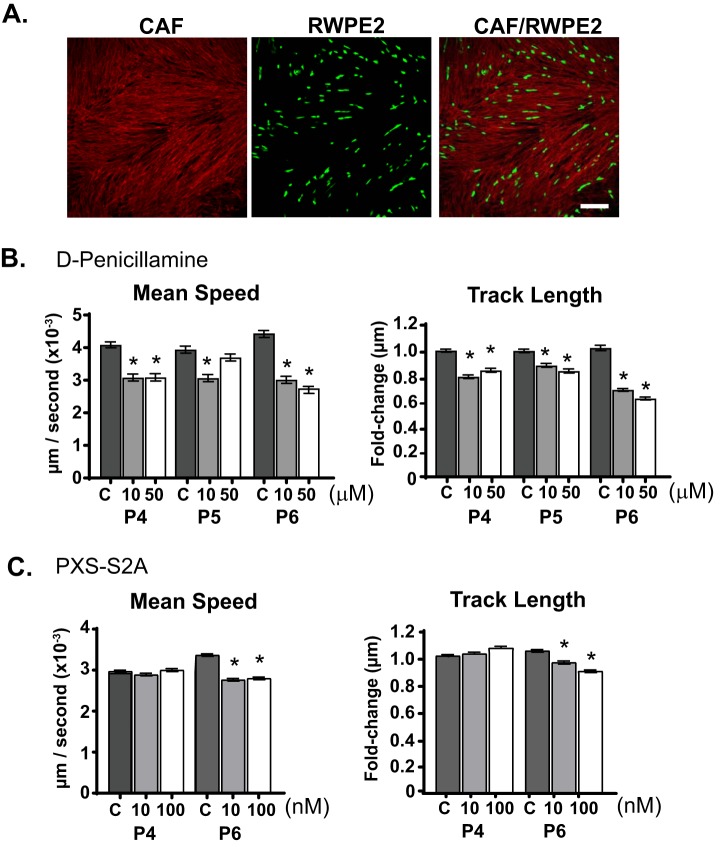
**LOX/LOXL2 inhibition in CAF impairs prostate epithelial cell motility**. *A*, Representative images of fluorescently-labeled transformed prostate epithelial cells (RWPE2; green) in co-culture with patient CAF stained with fibronectin (red). Scale bar = 50 μm. CAF cultures were pre-treated with (*B*) 10 or 50 μm of d-Penicillamine (Patient 4–6), *C*, 10 and 100 nm of PXS-S2A (Patient 4 and 6) or vehicle control (*C*) for 24 h before drug wash-out. RWPE2 migration was assessed over 12 h in CAF co-cultures and presented as mean speed and track length (normalized to control CAF; C). Bars represent the mean ± S.E. (*n* = 3). *p value <0.05 compared with control CAF.

To characterize the role of autocrine LOXL2 signaling in regulating CAF motility, CAF were subjected to a wound healing assay (Fig. 9*A*) in the presence or absence of the LOX/LOXL2 inhibitor, DPEN or the specific LOXL2 inhibitor PXS-S2A (Fig. 9). DPEN treatment at 50 μm, but not 10 μm, significantly impaired CAF migration in all three patients (Fig. 9*B*) whereas PXS-S2A treatment impaired CAF migration at both 10 nm and 100 nm concentrations (Figs. 9*C*). Because CAF also regulate the migration ability of prostate tumor epithelium (20), we next assessed the potential paracrine role of LOX/LOXL2 secreted by CAF. CAF were pre-treated with DPEN or PXS-S2A, followed by inhibitor wash-out, and then co-cultured with the transformed prostate epithelial cell line RWPE-2 (56) for a further 12 h (Fig. 10*A*). Pre-treatment of CAF with the LOXL2 inhibitor DPEN significantly impaired the mean speed and track length of co-cultured RWPE-2 prostate tumor cells in all patients tested (Patient 4–6; Fig. 10*B*). PSX-S2A-treated CAF (at both 10 and 100 nm concentrations) inhibited prostate tumor cell migration in Patient 6, whereas Patient 4 CAF co-cultures did not show a response (Fig. 10*C*). These data indicate a degree of inter-patient heterogeneity in the functional role of specific LOX/LOXL enzymes.

Combined, these data indicate that LOX/LOXL2 production by prostate CAF produces a highly aligned ECM that can potentiate the migration of CAF themselves, as well as neighboring prostate tumor cells.

## DISCUSSION

CAF-derived ECM plays a critical role in promoting cancer dissemination (57) and represents an emerging therapeutic target in solid tumors (58). The presence of an “altered” or reactive stroma is associated with poor patient prognosis in prostate (6, 59) and other solid cancers (60, 61); however, the underlying stromal-epithelial signal networks remain poorly characterized. Here we applied an HRM-DIA mass spectrometry (MS)-based proteomic strategy combined with primary human patient-matched prostatic CAF and NPF to resolve key mediators of intercellular signaling within the tumor stroma. We present the first data showing that the inhibition of LOXL2 in prostate CAF can significantly perturb the prostate tumor microenvironment.

Traditionally, global proteomics has been performed utilizing data-dependent acquisition (DDA) MS methods for protein identification and quantification. In DDA, a survey scan (MS1) selects a subset of peptide precursors for subsequent fragmentation acquiring fragment-ion spectra (MS2). Quantitation is then mapped back to the MS1 peak. This approach is well known to be biased and stochastic, with reproducibility becoming problematic with complex samples (62, 63). Within the last decade, combined efforts in the MS community have pioneered quantification directly from MS2 spectra utilizing data-independent acquisition (DIA), to bypass the limitations associated with DDA (64–67). Here, targeted extraction of quantitative information for peptides is enabled through use of a reference spectral library. Using a proteomic strategy based on this concept, our global proteomic analysis of CAF established from primary prostate tumors identified a prominent protein-protein interaction hub centered on the DDR2-collagen-LOXL2 signaling axis, critical for ECM remodeling and function (62, 68, 69). Although the focus of this study was not to specifically investigate ECM proteins, an important role was evident and further investigation using enrichment for this and other specific subcellular compartments (70, 71) could refine our dataset and further contribute to our understanding of the role of matricellular proteins in tumor progression.

Aberrant collagen deposition, topography and composition is a cornerstone of solid tumor development (72). In prostate cancer, microscale biomechanical analyses of tissue biopsies have revealed that collagen orientation and tissue stiffness are increasingly dysregulated with increasing tumor grade (73). In other cancers, fibrillar collagen and its receptor, DDR2, work in concert to promote a pro-tumorigenic environment via both the tumor microenvironment and tumor cells (74). For example, CAF-derived collagen can activate DDR2 signaling in the epithelium, promoting proliferation and invasion by upregulating regulators of epithelial-to-mesenchymal transition (EMT) such as SNAI1 (75), leading to metastasis (74). Concurrently, activation of CAF DDR2 can activate downstream programs that alter ECM architecture, resulting in a stiffer, desmoplastic, linearized matrix (74–76). This biomechanically aberrant environment may in turn promote further migration of cancer epithelial cells (77, 78). Collectively, the up-regulation of CAF-derived collagen and DDR2 during early prostate cancer may potentiate a pro-tumorigenic loop and drive disease progression.

Although LOXL2 has been implicated in the progression of multiple solid tumors (54) and is overexpressed in prostate cancer (53), few studies have reported a specific role for LOXL2 in prostate CAF function (40, 79). Here we demonstrate that prostate CAF have significantly higher LOXL2 enzymatic activity compared with matched NPF. We also show a cell-autonomous role of LOX/LOXL, and more specifically, LOXL2 in promoting CAF motility and ECM organization. We demonstrate that prostate CAF produce a highly aligned ECM network compared with NPF, on which prostate tumor epithelium migrated extensively. To further dissect the specific role of the ECM, and assess whether LOXL2-regulation of the ECM is sufficient to mediate the biological effects of this enzyme, we attempted to decellularize our primary prostate CAF and NPF cultures (80). Unfortunately, inefficient ECM retention following decellularization of primary human CAF precluded downstream analyses.

Pharmacological inhibition of LOX/LOXL2 abrogated the ECM network of CAF and significantly impeded tumor cell migration, like recent findings of CAF-LOXL2 function in breast cancer xenografts (81) and human gastric cancer cell lines (79). Notably, there was some inter-patient variation observed in protein expression and the functional response to LOX/LOXL2 inhibition. This is reminiscent of several other differentially expressed genes in CAF (14) and warrants further analysis to determine whether LOXL2 levels may be associated with the clinic-pathologic or genomic features of the tumor.

Tumor-secreted LOXL2 activates CAF through FAK signaling (51), and this may occur via enhanced ECM stiffness (54). Increased phosphorylation of FAK at Y925 was observed within our prostate CAF compared with patient-matched NPF, indicating that this may represent a common microenvironmental signaling pathway across different solid tumor types (51, 54). Further, a recent study has shown that FAK is up-regulated during prostate cancer progression and promotes resistance to chemotherapy (82), supporting the use of concomitant FAK inhibitors with standard-of-care treatment. Similarly, next generation LOXL2 inhibitors are effective in pre-clinical models of gastric (83) and breast cancer (40, 84, 85) as well as lung and liver fibrosis models (86), with targeted therapeutics against LOXL2 currently undergoing Phase I/II testing (54). It is evident that dysregulation of the DDR2-collagen-LOXL2 axis in early prostate cancer would therefore provide a potent and pro-tumorigenic microenvironment, capable of driving aggressive disease and that this axis can be targeted with therapeutic interventions. Together our data highlights the role of prostate CAF in regulating the tumor microenvironment and importantly, identifies an underpinning molecular mechanism within the ECM.

Contact-mediated signaling complexes enable fibroblasts to drive cancer cell invasion via the stromal cytoskeleton (87, 88), although little is known of the underlying mechanism. In our proteomic and phosphoproteomic datasets, there was an enrichment for cytoskeletal related categories such as “Cell Adhesion,” “Focal Adhesion,” “Actin Binding,” and “Actin Cytoskeleton.” Previous studies have shown that CAF have increased levels of contractile actin as well as stress fibers and focal adhesions (89, 90). Functionally, this up-regulation can lead to tissue stiffness, resulting in subsequent pro-tumorigenic mechano-transduction signaling in the tumor microenvironment (91). For the first time in prostate CAF we identified a prominent hub involving the phosphorylation of serine/threonine kinases: T326-PAK6, S2-PAK2, and S386-MAPK6. Previous studies have shown that p21-activated kinases (PAKs) are oncogenic drivers (92) and regulate actin organization in mammalian cells (93), with MAPK6 having been identified as a substrate for PAK2 (94). Collectively, our data indicate that the stromal cytoskeleton may play an important role in cancer cell-CAF signaling, and facilitate both stromal and epithelial invasion.

## CONCLUSION

The activation of CAF within solid tumors plays an ongoing role in tumorigenesis and provides an important source of potential therapeutic targets (74, 95, 96). The autocrine and paracrine effects induced by LOXL2 that include, but are not limited to, tumor cell proliferation and invasion, fibroblast activation, ECM remodeling, increased angiogenesis, and promotion of EMT renders LOXL2 inhibition an attractive therapeutic strategy that targets both tumor cells and the surrounding stroma. Our data provide significant insight into the mechanisms underlying stromal function during early prostate tumorigenesis that can promote aggressive disease.

## DATA AVAILABILITY

The MS proteomic data have been deposited to the Mass spectrometry Interactive Virtual Environment (MassIVE) consortium (http://www.massive.ucsd.edu/ProteoSAFe/datasets.jsp) with data set identifier: MSV000082309. Data interpretation of the whole proteome spectral library with .RAW files, result file, and the MaxQuant software version has been deposited to the Proteome Xchange under the Pride identifier PXD010611. Phosphoproteome data interpretation has been deposited to MassIVE consortium data set identifier: MSV000083735.

## Supplementary Material

supplemental Fig. S2

Supporting Information Table Legend

Supporting Information

Supplemental Figure Legends

Supplemental Figures

## Footnotes

^1^ The abbreviations used are:

CAF: cancer-associated fibroblasts
ACN: acetonitrile
AGC: automatic gain control
bFGF: basic fibroblast growth factor
DDA: data-dependent acquisition
DDR2: discoidin domain-containing receptor 2
DFS: disease-free survival
DIA: data-independent acquisition
DPEN: D-penicillamine
ECM: extracellular matrix
EMT: epithelial-to-mesenchymal transition
FA: formic acid
HI-FBS: heat inactivated fetal bovine serum
HRM: hyper-reaction monitoring
iRT: indexed Retention Time
IT: ion trap
LOX: lysyl oxidase
LOXL2: lysyl oxidase-like 2
LC-MS/MS: liquid chromatography-tandem mass spectrometry
NPF: non-malignant prostate fibroblast
PCA: principal component analysis
TFA: trifluoroacetic acid
RP: radical prostatectomy
RPMI: roswell park memorial institute.
