# Supplementary material for: Proteomic Profiling of Human Prostate Cancer-associated Fibroblasts (CAF) Reveals LOXL2-dependent Regulation of the Tumor Microenvironment
Source: Mol Cell Proteomics. 2019 May 6;18(7):1410–27. doi: 10.1074/mcp.RA119.001496 (PMC6601211; doi:10.1074/mcp.RA119.001496)
Supplement: supplemental Fig. S2 [file RA119.001496_index.html]

Supplement to Proteomic profiling of human prostate cancer-associated fibroblasts (CAF) reveals LOXL2-dependent regulation of the tumor microenvironment | Molecular & Cellular Proteomics

## Supplemental Data

- Supporting Information Table Legend - Supporting Information Table Legend
- Supporting Information - Supporting Information
- Supplemental Figure Legends - Supplemental Figure Legends
- Supplemental Figures - Supplemental Figures
