# Supplementary material for: Proteomic Profiling of Human Prostate Cancer-associated Fibroblasts (CAF) Reveals LOXL2-dependent Regulation of the Tumor Microenvironment
Source: Mol Cell Proteomics. 2019 May 6;18(7):1410–27. doi: 10.1074/mcp.RA119.001496 (PMC6601211; doi:10.1074/mcp.RA119.001496)
Supplement: Supporting Information Table Legend [file 144877_1_supp_323407_pqssjg.pdf]

Supplemental Table Legends

Supplemental Table 1: Clinicopathological features of tumors of origin for cultured fibroblast cell lines.

Key: TNM = tumour, (lymph) node, metastasis; EPE = extraprostatic extension; SV = seminal vesicle; CK8/8 = cytokeratin 8/18; HMWCK = high molecular weight cytokeratin; NPF = non-malignant prostate fibroblasts; CAF = cancer-associated fibroblasts; ND = Not done.

Supplemental Table 2: Whole Proteome Spectral Library

(A) Peptides, # of unique peptides, intensities, and spectral count of each protein identified with DDA acquisition. **(B)** CV of proteome of individual samples across replicates (calculated from raw (unlogged value)).

Supplemental Table 3: Functional analysis of proteins identified in one or two CAF or NPF lines compared to three or more corresponding cell lines

Functional analysis of common proteins identified in **(A)** three or more patient CAF cell lines, **(B)** only one or two patient CAF cell lines, **(C)** three or more patient NPF cell lines, and **(D)** only one or two patient NPF cell lines.

Supplemental Table 4: Proteins Identified in non-enriched Samples

(A) 4075 proteins identified. **(B)** Top 3 proteotypic peptides requiring at least 3 transitions used in protein quantification.

Supplemental Table 5: Quantifiable Proteins Identified in individual non-enriched samples (3 biological replicates)

(A) 4075 quantifiable proteins based on peptide intensities passing criteria (log2 sum of the intensities of the top 3 proteotypic peptide for each protein). **(B)** Normalization of individual non-enriched samples (3 biological replicates). **(C)** CV of proteome of individual samples across replicates (calculated from raw (unlogged value)).

Supplemental Table 6: Unique phosphopeptides identified in TiO2-enriched Samples

(A) Unique Phosphorylated Peptides identified in TiO2 enriched samples. **(B)** Fragment ions of identified phosphorylated peptides used for quantitation.

Supplemental Table 7: Quantifiable phosphopeptide expression identified in individual TiO2-enriched samples (pool of 3 biological replicates)

(A) 12,209 quantifiable proteins based on peptide intensities passing criteria (log2 intensities). **(B)** Normalization of individual TiO2-enriched samples.

Supplemental Table 8: Significantly differentially expressed proteins between CAF and NPF.

(A) 363 differential proteins (raw P-value ≤.02). Differential proteins with higher stringent criteria (adjusted P-value <.05, FC>1.5) are highlighted in the right panel (grey shading). **(B)** Plot of local FDR estimation of 4075 proteins (generated by LocalFDR from Anapuce R package).

Supplemental Table 9: Phosphopeptides with significant differences in abundance between CAFs and NPFs (raw P-value <.01, FC>1.5)

(A) 161 differential phosphopeptides. **(B)** Plot of local FDR estimation of 12,209 phosphopeptides (generated by LocalFDR from Anapuce R package).
