# Supplementary material for: Proteomic Profiling of Human Prostate Cancer-associated Fibroblasts (CAF) Reveals LOXL2-dependent Regulation of the Tumor Microenvironment
Source: Mol Cell Proteomics. 2019 May 6;18(7):1410–27. doi: 10.1074/mcp.RA119.001496 (PMC6601211; doi:10.1074/mcp.RA119.001496)
Supplement: Supplemental Figure Legends [file 144877_1_supp_323410_pqssjg.pdf]

Supplemental Figure Legends

Supplemental Figure 1. Interactions of proteins exhibiting differential phosphorylation using STRING software.

(A) Interactome for proteins with increased phosphorylation in CAF compared to NPF. Differentially phosphorylated sites are indicated**. (B)** Interactome for proteins with decreased phosphorylation in CAF compared to NPF. Differentially phosphorylated sites are indicated.

Supplemental Figure 2. Expression of CD90 and CD166 antigen in individual patient CAF/NPF by flow cytometry.

(A) Representative flow plots show CD90 and CD166 surface antigen expression in matched NPF/CAF from Patient 1-6 (P1-6) compared to unstained controls (cells are gated on propidium iodide to exclude dead cells). **(B)** Graph shows the percentage of CD90 and CD166 double positive cells in NPF and CAF from P1-6. Bars show mean ± SEM (n=3). Related to Figure 6A-B.

Supplemental Figure 3. Replicate western blot and densitometry

(A) Biological replicate western blot images for LOXL2 and β-actin/α-tubulin loading controls from matched pairs of NPF (N1-6) and CAF (C1-6) derived from Patients 1-6; 20 μg of protein was loaded per lane. We note that LOXL2 is detected in P3 samples in this blot, but not in Figure 6C. The reason for this is unclear, but may reflect the difference in passage number of the biological samples. **(B)** Graphs show quantification of protein expression levels by densitometry for LOXL2 protein relative to α-tubulin or β-actin. Bars represent the mean ± SEM (n= 6 patients); *P-

value <0.05. **(C)** Quantification of pFAK Y925 relative to total FAK protein by densitometry for biological replicate. FAK and pFAK protein expression was first quantified relative to their respective β-actin loading controls. Bars represent the mean ± SEM (n= 6 patients); *P-value <0.05. Biological replicates are derived from different passages of CAF/NPF. Related to Figure 6.

Supplemental Figure 4. LOXL2 uncropped western blots

Replicate uncropped western blot images for **(A)** LOXL2 and α-tubulin loading controls (related to Figure 6C) and **(B)** LOXL2 and β-actin/α-tubulin loading controls (related to Supplementary Figure 3). Biological replicates are derived from different passages of CAF/NPF.

Supplemental Figure 5. DDR2 and FAK and pFAK Y925 uncropped western blots

(A) Uncropped western blot images for DDR2 and β-actin loading control (related to Figure 6D). **(B)** Uncropped western blot images for FAK and pFAK Y925 protein and β-actin loading controls (related to Figure 6E).

Supplemental Figure 6. Reduced disease free survival in prostate cancer patients with high LOXL2 expression.

Kaplan-Meier plot shows worse disease-free survival in patients with high LOXL2 expression (hazard ratio 1.506; 95% confidence intervals 0.99-2.29; P-value 0.048) based on gene expression and clinical information derived from the National Cancer

Institute GDC Data Portal containing 492 patients RNA-seq data. Patients were stratified to high and low LOXL2 gene expression using a cut-off of 0.6. Disease-free survival (DFS) was plotted using the Kaplan-Meier curve and differences in DFS were evaluated using the log-rank statistical test.

Supplemental Figure 7. ECM staining in prostate NPF and CAF.

(A) Raw images (top panels) and processed images (bottom panels) show respective fibronectin/orientation staining in NPF and CAF from **(i)** Patient 4 and **(ii)** Patient 6, following treatment with 10 and 50 µM D-penicillamine or 10 and 100 nM of PXS-S2A. Scale bar = 50 µm. Images in bottom panels were processed and colour-coded to represent the degree of fibre orientation distribution within each sample. **(B)** Raw images show individual fibronectin and collagen I staining or composite confocal image (fibronectin/collagen I). Images are representative (n=3). Scale bar = 50 µm. Related to Figure 8.
