# Supplementary material for: Proteomic Profiling of Human Prostate Cancer-associated Fibroblasts (CAF) Reveals LOXL2-dependent Regulation of the Tumor Microenvironment
Source: Mol Cell Proteomics. 2019 May 6;18(7):1410–27. doi: 10.1074/mcp.RA119.001496 (PMC6601211; doi:10.1074/mcp.RA119.001496)

## Supplemental Figure 1

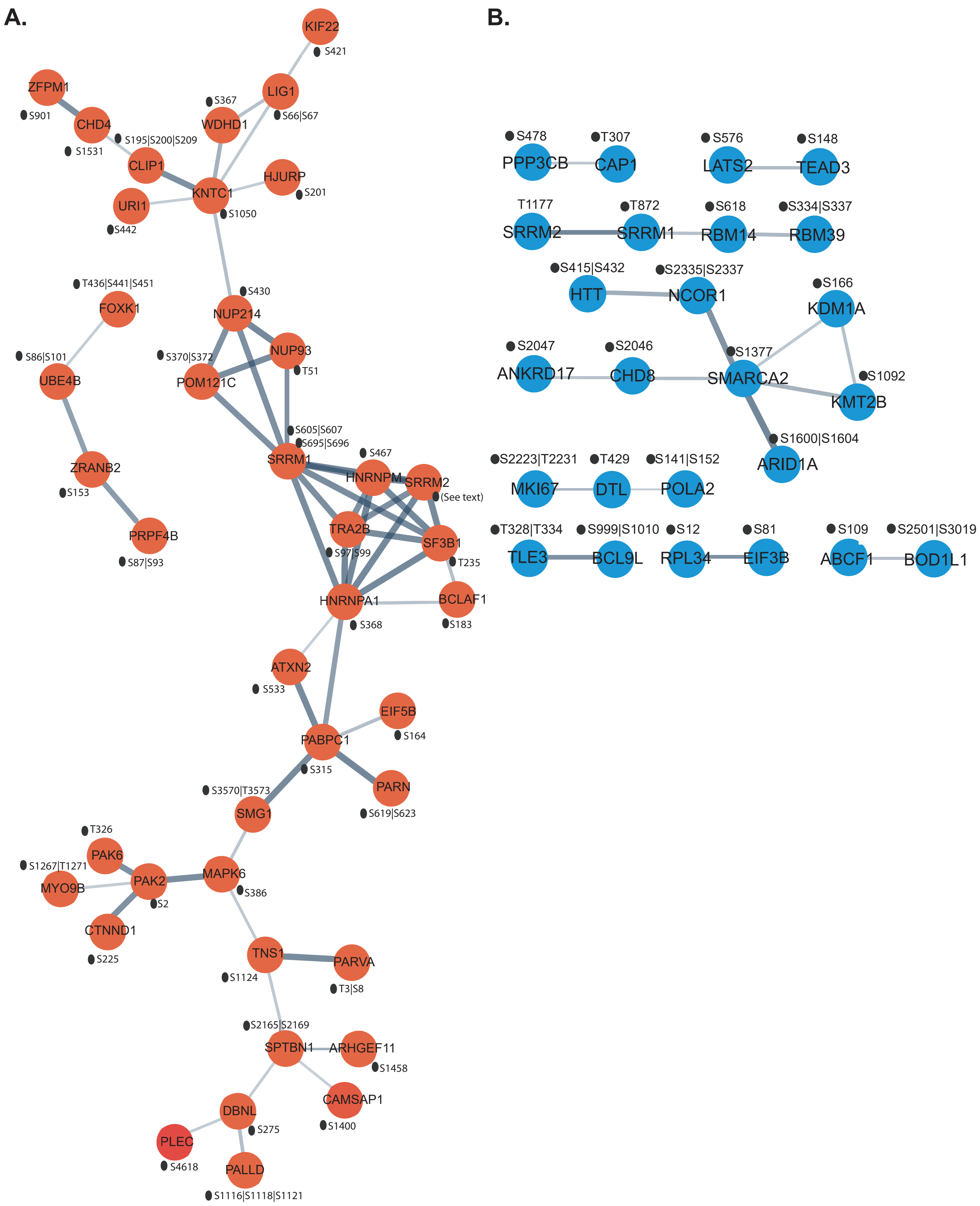

**Supplemental Figure 2**

A.

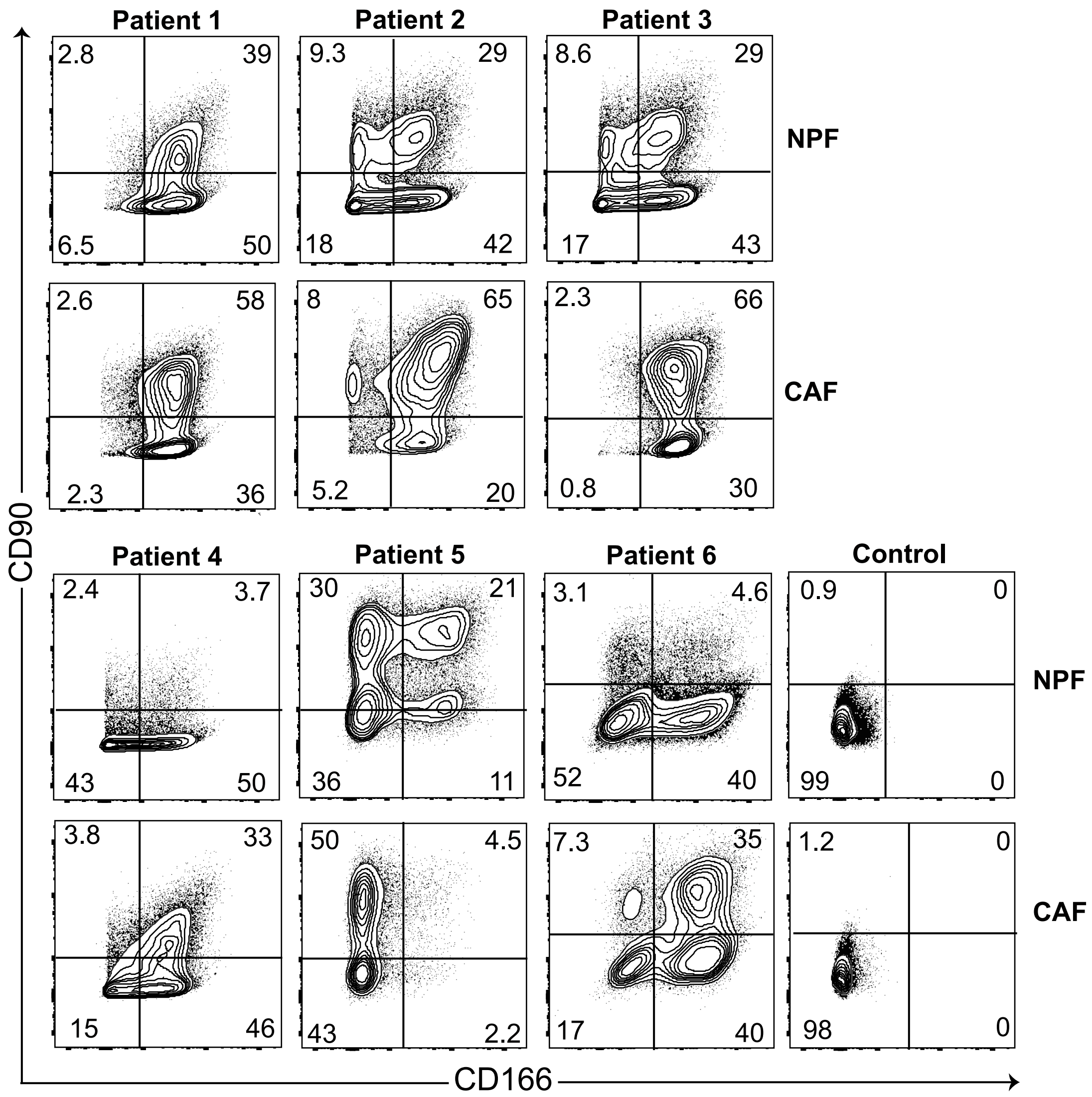


B.

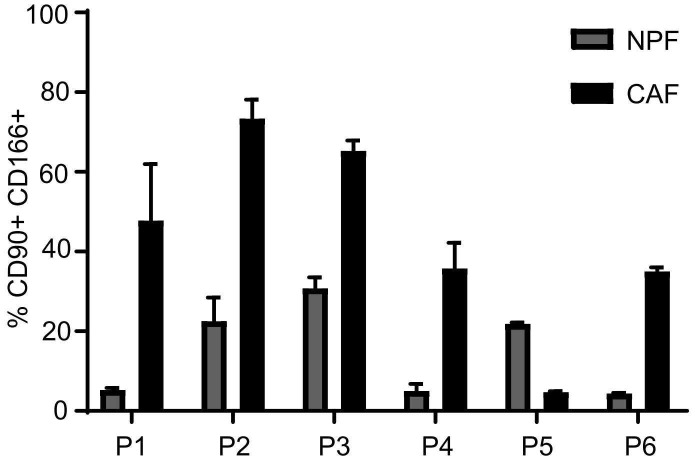

## Supplemental Figure 3

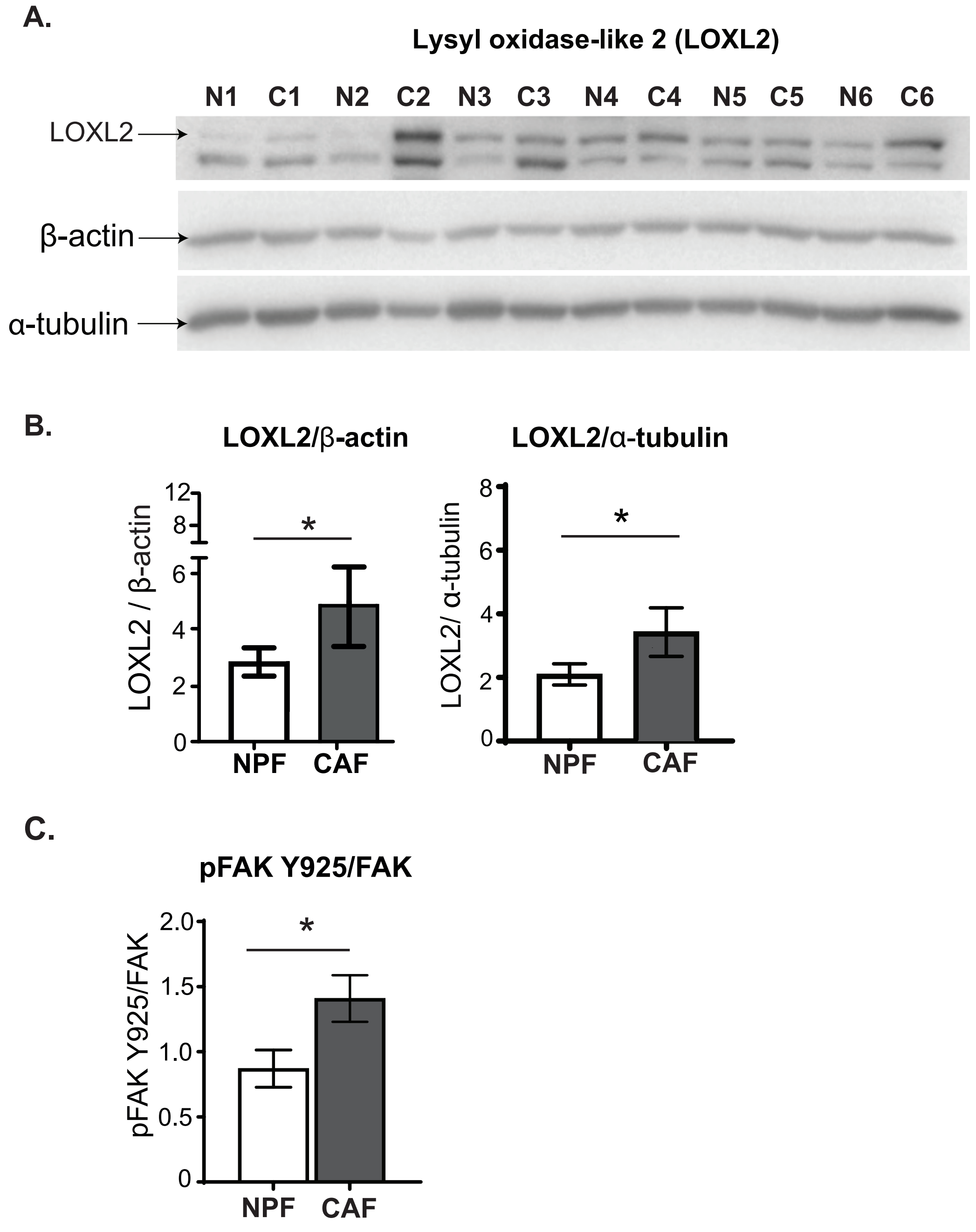

## Supplemental Figure 4

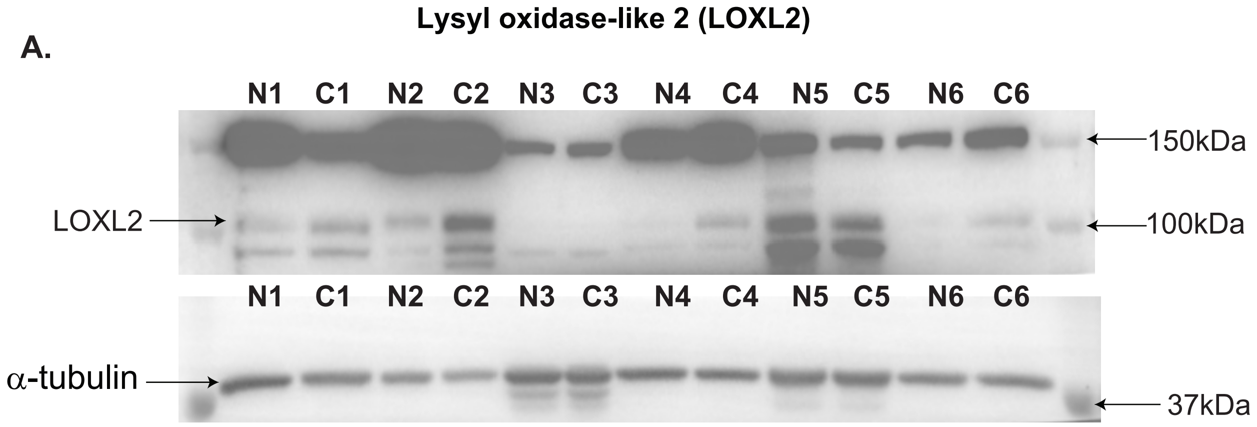


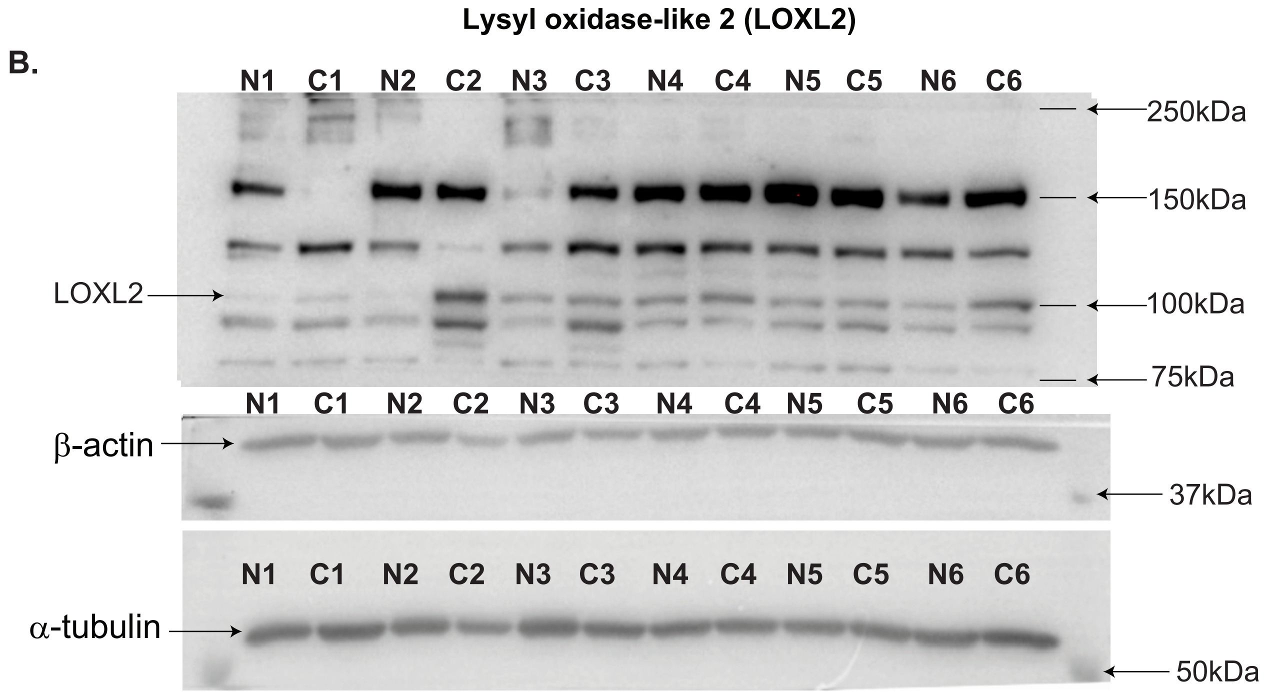

## Supplemental Figure 5

**A.**

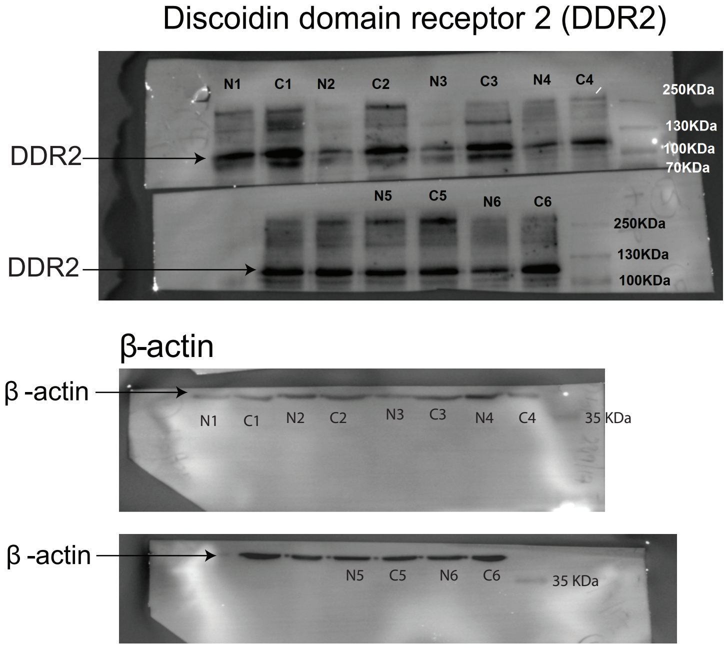


**B.**

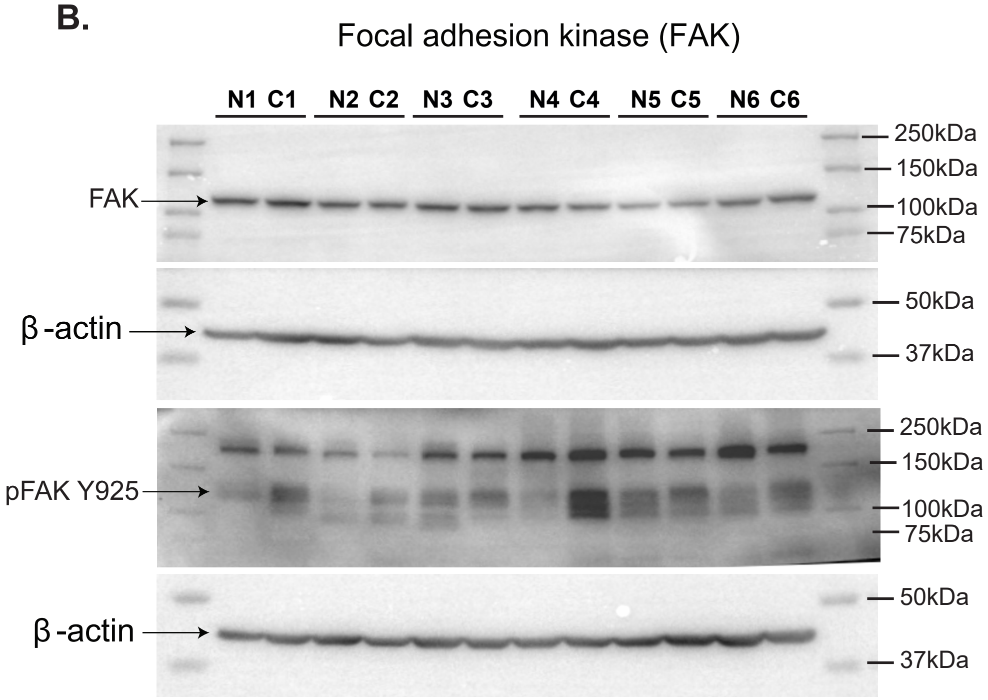

# Supplemental Figure 6

A.

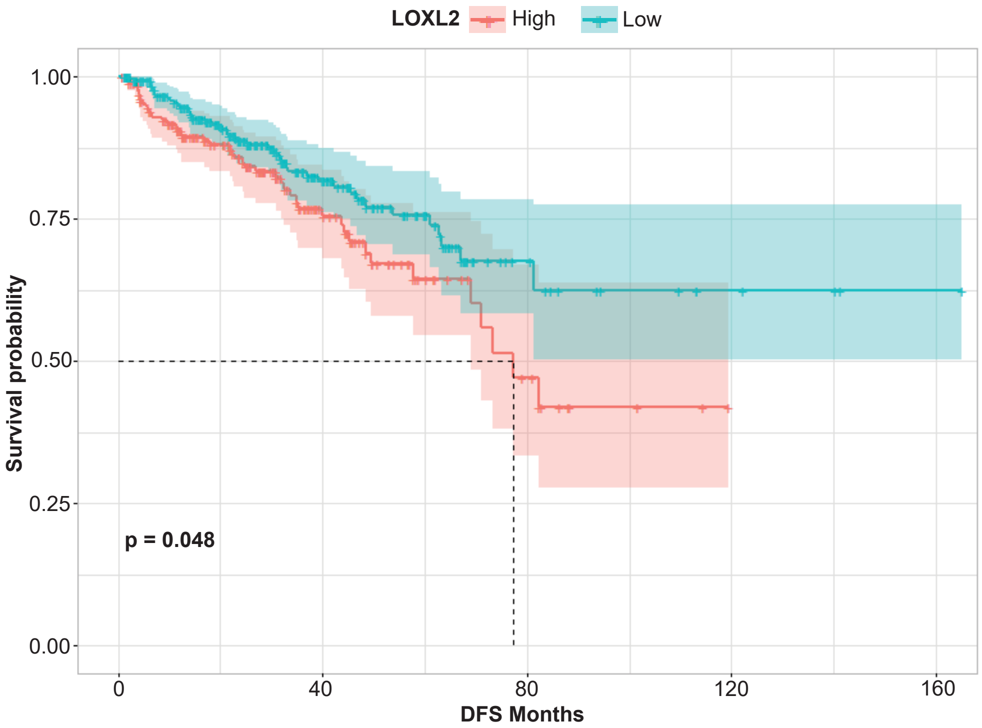

# Supplemental Figure 7

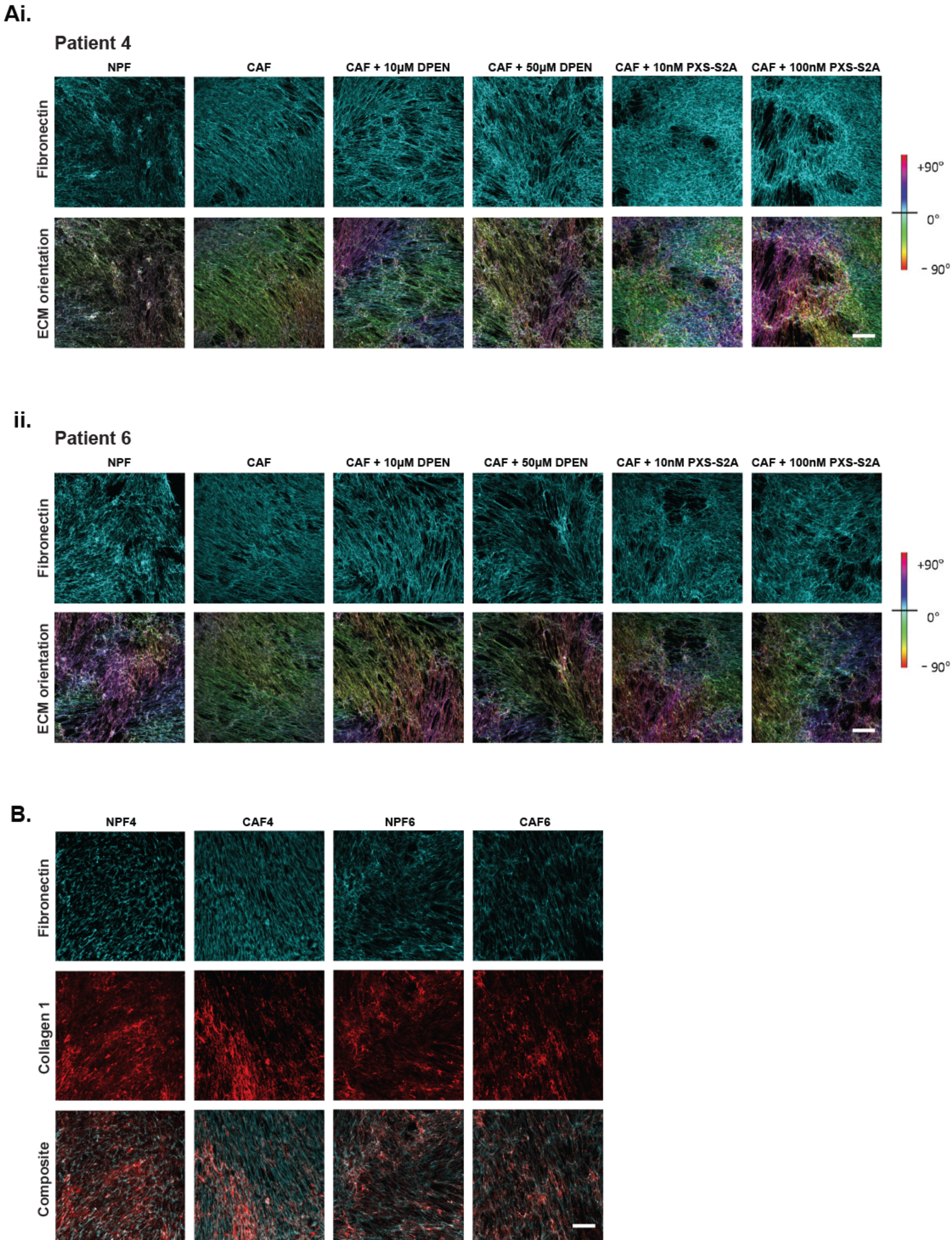

Supplement: Supplemental Figures [file 144877_1_supp_323413_pqssjg.pdf]
